# Association Mapping for Fruit, Plant and Leaf Morphology Traits in Eggplant

**DOI:** 10.1371/journal.pone.0135200

**Published:** 2015-08-18

**Authors:** Ezio Portis, Fabio Cericola, Lorenzo Barchi, Laura Toppino, Nazzareno Acciarri, Laura Pulcini, Tea Sala, Sergio Lanteri, Giuseppe Leonardo Rotino

**Affiliations:** 1 Dipartimento di Scienze Agrarie, Forestali ed Alimentari (DISAFA)—Plant Genetics and Breeding, University of Torino, I-10095 Grugliasco, Torino, Italy; 2 Consiglio per la ricerca in agricoltura e l’analisi dell’economia agraria—CREA, Research Unit for Vegetable Crops, I-26836 Montanaso Lombardo, Lodi, Italy; 3 Consiglio per la ricerca in agricoltura e l’analisi dell’economia agraria—CREA, Research Unit for Vegetable Crops, I-63030 Monsampolo del Tronto, Ascoli Piceno, Italy; University of Tsukuba, JAPAN

## Abstract

An eggplant (*Solanum melongena*) association panel of 191 accessions, comprising a mixture of breeding lines, old varieties and landrace selections was SNP genotyped and phenotyped for key breeding fruit and plant traits at two locations over two seasons. A genome-wide association (GWA) analysis was performed using the mixed linear model, which takes into account both a kinship matrix and the sub-population membership of the accessions. Overall, 194 phenotype/genotype associations were uncovered, relating to 30 of the 33 measured traits. These associations involved 79 SNP loci mapping to 39 distinct chromosomal regions distributed over all 12 eggplant chromosomes. A comparison of the map positions of these SNPs with those of loci derived from conventional linkage mapping showed that GWA analysis both validated many of the known controlling loci and detected a large number of new marker/trait associations. Exploiting established syntenic relationships between eggplant chromosomes and those of tomato and pepper recognized orthologous regions in ten eggplant chromosomes harbouring genes influencing breeders’ traits.

## Introduction

In terms of production, eggplant (*Solanum melongena* L.) (also referred to as aubergine or brinjal) is the third most important Solanaceous crop after potato and tomato [1]. It provides a reliable source of minerals, vitamins and antioxidants to the human diet. While sharing many of the breeding goals relevant to other fruit crops (primarily yield, resistance/tolerance to biotic and abiotic stress and long shelf life), some important breeders' traits are highly specific to eggplant–in particular the need to reduce fruit bitterness, leaf and fruit calyx prickliness. Despite the economic importance attached to eggplant improvement, its genome is less well explored than those of its closely related Solanaceous species tomato, potato and pepper. As a result, marker assisted selection has not yet been widely adopted by eggplant breeders.

The genetic basis of certain fruit and plant morphology traits has been identified by linkage mapping based on both intra-specific [2] and inter-specific [3–5] populations. In a pioneering attempt to apply a genome-wide association (GWA) approach, Ge et al. [6] were able to identify a number of phenotype/genotype associations related to eight fruit-related traits. The GWA approach has certain advantages over biparental linkage mapping. It allows for a much wider sampling of phenotypic and genotypic variation than is possible when a choice of just two lines (the parents of the biparental cross) is required. Furthermore, it exploits the fact that the accessions will have experienced multiple rounds of recombination, in contrast to the few possible during the construction of a mapping population. Finally, it can incorporate numerous accessions of direct relevance to crop improvement [7,8]. In a previous SNP-based study of an eggplant association panel, it was demonstrated that linkage disequilibrium was sufficiently high to allow for an efficient coverage of the genome with just a moderate number of markers [9]. The same SNP data were also effective in identifying 56 genomic regions harbouring genes affecting anthocyanin pigmentation and distributed over nine of the 12 eggplant chromosomes. Here, the same association panel and SNP data set has been used to identify and position marker/trait associations related to fruit, plant and leaf morphological traits relevant for eggplant breeding.

## Material and Methods

### Permission

No specific permits were required for the described field studies, which took place in two experimental fields at the CRA-ORL in Montanaso Lombardo and CRA-ORA in Monsampolo del Tronto (Italy). These field plots were used by the authors of this paper affiliated to the aforementioned institution (LT, NA, LP, TS and GLR) for phenotypic characterization of the eggplant population.

### Plant material and the evaluation of phenotype

The set of 191 accessions making up the germplasm panel (S1 Table) included representatives of breeders' lines, old varieties and landraces, and is identical to the one described by Cericola et al. [10]. The whole panel was grown in field at two sites (Montanaso Lombardo (ML) 45°20'N, 9°26'E and Monsampolo del Tronto (MT) 42°53'N, 13°47'E) over two consecutive seasons in two completely randomized blocks with six plants per accession per block. Standard horticultural practices were applied. The 33 chosen traits (relating to either fruit morphology or plant and leaf morphology, and listed in Table 1) were based on descriptors defined by the European Cooperative Programme for Plant Genetic Resources Solanaceae (ECPGR [11]) and the International Board for Plant Genetic Resource (IBPGR [12]). The traits assayed are the following:

**Table 1:** Codes used to identify the traits measured, along with statistics describing their variation among the members of the association panel.

Trait	Code	Average	SD	CV	Min	Max	h^2^
***Fruit-related traits*:**							
Fruit weight (g)	*fw*	256.97	122.26	0.48	8.5	1750	0.48
Fruit length (cm)	*fl*	14.39	5.53	0.38	0.5	48.1	0.76
Fruit diameter 1/4 (cm)	*fd1/4*	6.09	2.42	0.40	0.6	25.0	0.75
Fruit diameter 1/2 (cm)	*fd1/2*	7.11	2.66	0.37	1.1	28.1	0.74
Fruit diameter 3/4 (cm)	*fd3/4*	6.74	2.17	0.32	1.0	23.5	0.73
Fruit diameter max (cm)	*fdmax*	7.39	2.59	0.35	1.3	28.1	0.72
Fruit diameter max position (scale 1–8)	*fdmaxp*	5.84	0.72	0.12	3	8	0.38
Fruit shape	*fs*	2.49	1.88	0.76	0.1	13.8	0.89
Fruit curvature (scale 1–9)	*fcur*	2.08	1.38	0.66	1	9	0.54
Fruit apex shape (scale 3–7)	*fas*	4.70	0.95	0.20	3	7	0.46
Peduncle length (cm)	*pedl*	4.23	1.26	0.30	1.3	14.5	0.53
Fruit calyx prickliness (scale 0–9)	*fcpri*	1.65	1.83	1.11	0	9	0.38
Fruit calyx removal (binary)	*fcr*	0.81	0.38	0.47	0	1	0.20
Calyx coverage (scale 1–5)	*cacov*	2.44	0.74	0.30	1	5	0.13
Outer fruit firmness (Kg/cm2)	*outfir*	9.43	3.06	0.32	0.8	28.8	0.12
Inner fruit firmness (Kg/cm2)	*intfir*	3.62	0.91	0.25	0.1	11.8	0.14
Number of locules (number)	*slon*	4.58	1.49	0.33	2.0	19.0	0.41
Flesh color (binary)	*flcol*	0.23	0.24	1.04	0	1	0.15
Flesh green ring (binary)	*gring*	0.72	0.45	0.62	0	1	0.87
***Plant/leaf morphology-related trait*:**							
Plant growth habit (scale 1–9)	*hab*	3.83	1.62	0.42	1	9	0.64
Number of branches (number)	*br*	3.03	1.22	0.40	0	9	0.22
Leaf width (cm)	*lw*	11.56	1.96	0.17	5.0	24.4	0.39
Leaf length (cm)	*lle*	17.20	2.16	0.13	9.4	28.4	0.41
Adaxial leaf central venation prickl.(scale 0–5)	*adlcevepri*	0.43	0.65	1.53	0	5	0.21
Adaxial leaf lateral venation prickl. (scale 0–5)	*adllavepri*	0.11	0.41	3.77	0	5	0.54
Abaxial leaf central venation prickl. (scale 0–5)	*ablcevepri*	0.39	0.65	1.68	0	5	0.55
Abaxial leaf lateral venation prickl. (scale 0–5)	*abllavepri*	0.08	0.41	4.94	0	5	0.87
Stem prickliness (scale 0–5)	*stpri*	0.32	0.58	1.78	0	5	0.55
Abaxial leaf prickles number	*ablprin*	0.59	1.25	2.11	0	13	0.62
Adaxial leaf prickles number	*adlprin*	0.81	1.51	1.86	0	12	0.60
Leaf hairiness (scale 0–5)	*lha*	1.91	1.15	0.60	0	5	0.55
Number of flowers / inflorescence	*flwin*	1.94	0.95	0.49	1.0	11.4	0.57
Flowering time (number of days)	*flwt*	86.43	5.97	0.07	69	98	0.55

(SD, standard deviation; CV, coefficient of variation; Min, minimum; Max, maximum; h^2^ broad sense hereditability).

Fruit morphology traits were measured from five representative fruits per replication and comprised: fruit weight (*fw*), fruit length (*fl*), the diameter sampled in four parts of the fruit (*fd1/4*, *fd1/2*, *fd3/4* and *fdmax*), fruit diameter max position (*fdmaxp*; using a scale from 1 = close to the calyx to 8 = close to the apex), fruit shape (*fs*; the ratio between *fl* and *fdmax*), fruit curvature (*fcur*; 1 = no curvature; 3 = slightly curved, 5 = curved; 7 = S shaped, 9 = U shaped), fruit apex shape (*fas*; 3 = protruding apex shape; 5 = smooth apex shape; 7 = depressed apex shape), peduncle length (*pedl*), calyx prickliness (*fcpri*; 0 = no prickles, 1 = < 3 prickles, 3 = around 5 prickles 5 = around 10 prickles 7 = around 20 prickles 9 = > 30 prickles), calyx removal (*fcr*; describing the difficulty in detach the calyx from the fruit: 0: hard; 1: easy) and calyx coverage (*cacov*; using a scale from 1 = less than 10% of the fruit length to 5 = more than 50%). Peel and pulp resistance to mechanical penetration were measured by inserting a manual penetrometer; for peel firmness (*outfir*) assessed from a point halfway between the peduncle and the distal end of the fruit, while for pulp (*intfir*) was measured at the centre of a crosswise cut fruit. The fruit was cut transversely in the seed region to ascertain the number of seed locules (*slon*), as well as the flesh color (*flcol*) and presence/absence of green ring (*gring*) next to the skin.

The plant and leaf morphology traits were measured prior to the first harvest and comprised: growth habit (*hab*; scored on a scale from 1 = upright to 9 = prostrate), number of principal branches (*br*), leaf width (*lw*), leaf length (*lle*), leaf venation and stem prickliness (*adlcevepri*, *adllavepri*, *ablcevepri*, *abllavepri* and *stpri*; scored from 0 = no prickles to 5 = high number of prickles), leaf prickles number (*adlprin* and *ablprin*), leaf hairiness (*lha*; scale from 1 = no hairiness to 5 = high hairiness), the number of flowers per inflorescence (*flwin*; represented by the mean of five inflorescences), and the flowering time (*flwt*; defined by the day on which at least 50% of the plants within an accession displayed at least one opened flower).

### Statistical analysis and GWA mapping

The trait data were treated as adjusted accession means (best linear unbiased predictors). Several multivariate linear mixed models were tested using a combination of the F-test (for the fixed component) and the Akaike test (for the random component). The best fit model was: *p*_*ijsb*_ *= l*_*j*_ *+ y*_*s*_ *+ r*_*bjs*_ *+ g*_*i*_ *+m*_*ij*_ *+ n*_*is*_ *+ e*_*ijs*_, where *p*_*ijsb*_ represented the phenotype of the *b* ^*th*^ replicate of the *i* ^*th*^ genotype at the *j* ^*th*^ location in the *s* ^*th*^ season; *l*_*j*_ the fixed effect of the *j* ^*th*^ location, *y*_*s*_ the fixed effect of the *s* ^*th*^ season, *r*_*bjs*_ the fixed effect of the *b* ^*th*^ replicate within the *j* ^*th*^ location in the *s* ^*th*^ season, *g*_*i*_ the random effect of the *i* ^*th*^ genotype, *m*_*ij*_ the random effect of the genotype by location interaction, *n*_*is*_ the random effect of the genotype by season interaction and *e* the residual. Broad sense heritabilities were calculated from the expression $h^{2}=\frac{{\sigma^{2}}_{g}}{{\sigma^{2}}_{g}+{\sigma^{2}}_{y}/n_{y}+{\sigma^{2}}_{l}/n_{l}+{\sigma^{2}}_{e}/\left(n_{y}\times n_{l}\right)}$, where *σ* ^2^_*g*_ represented the genotypic variance, *σ* ^2^_*y*_ the genotype x year interaction, *σ* ^2^_*l*_ the genotype x location interaction, *σ* ^2^_*e*_ the residual variance, *n*_*y*_ the number of years (2) and *n*_*l*_ the number of locations (2). Pearson correlation coefficients were calculated between each pair of traits. All analyses were carried out using software implemented in the *R* package [13].

The GWA analysis was performed using Tassel v4.0.25 software [14]. The genotypic data comprised the 314 SNP loci described by Cericola et al. [9], 307 of which have known locations on the Barchi et al. [15] genetic map. Three models were tested, namely the simple general linear model (GLM, Naive-model), the structured association model (GLM, Q-model) and the mixed linear model (MLM, K+Q-model) [16]. A cumulative density function was used to assess the efficiency of each model, correcting for population structure. The false positive rate (p-value) was converted into a false discovery rate [17], using the QVALUE package implemented in *R*. q-values <0.05 were considered as significant. For each SNP locus significantly associated with trait variation, a general linear model with all fixed effect terms was applied to estimate the proportion of the phenotypic variance explained (PVE). In order to visualize the associations and compare them with established genes or quantitative trait loci (QTL) [2–6,18], any SNPs associated with a given trait linked by less than twice the global linkage disequilibrium were considered as a single unit defining association groups. The resulting genetic map, which incorporates information captured by Barchi et al. [15], was drawn using MapChart v2.1 software [19]. Synteny between tomato and eggplant chromosomal regions was investigated by a BLAST search of RAD tag sequences [20] surrounding informative SNPs against the tomato SL2.40 genome sequence [21] and aligned using the Burrows-Wheeler alignment tool [22]. Alignments with a mapping quality value >10 were considered as valid.

## Results

### Phenotypic variation and inter-trait correlations

A summary of the accessions' phenotypic performance and the related broad sense heritabilities is presented in Table 1. PVEs are given in S1 and S2 Figs.

Fruit morphology: most of the traits were highly variable, and the genotypic contribution to the variance was substantial in most of the case. Among the quantitative traits, the most variable were *fs* and *fw*. The most highly heritable traits were *fl*, *fd1/4*, *fd1/2*, *fd3/4*, *fs* and *gring* (Table 1, S1 Fig). The least variable traits were *outfir*, *intfir*, *flcol*, *cacov* and *fcr*, and these were also the traits associated with the highest genotype x environment interaction. *fw*, *fd1/4*, *fd1/2*, *fd3/4*, *fdmax* and *slon* were strongly and positively correlated with one another, as were *fl*, *fs*, *pedl*, *fcur* and *fdmaxp*. Fruit weight-related traits were negatively correlated with fruit length and shape-related traits (S3 Fig).

Plant and leaf morphology: most of the traits were highly variable, and the genotypic contribution to the variance was substantial in each case (Table 1). The most variable traits were those related to leaf prickliness (*abllavepri*, *adllavepri* and *ablprin*). The traits associated with the highest heritabilities were *abllavepri*, *hab* and *adlprin*, while the least heritable were *adlcevepri* and *br*. Both *br* and the *adlcevepri* were the traits associated with the highest genotype x environment interaction. The range in performance for each trait is shown in S4 Fig. The trait *lw* was highly and positively correlated with *lle*, as were the traits related to prickliness with those related to prickle number.

### Association mapping

**Fig 1:**
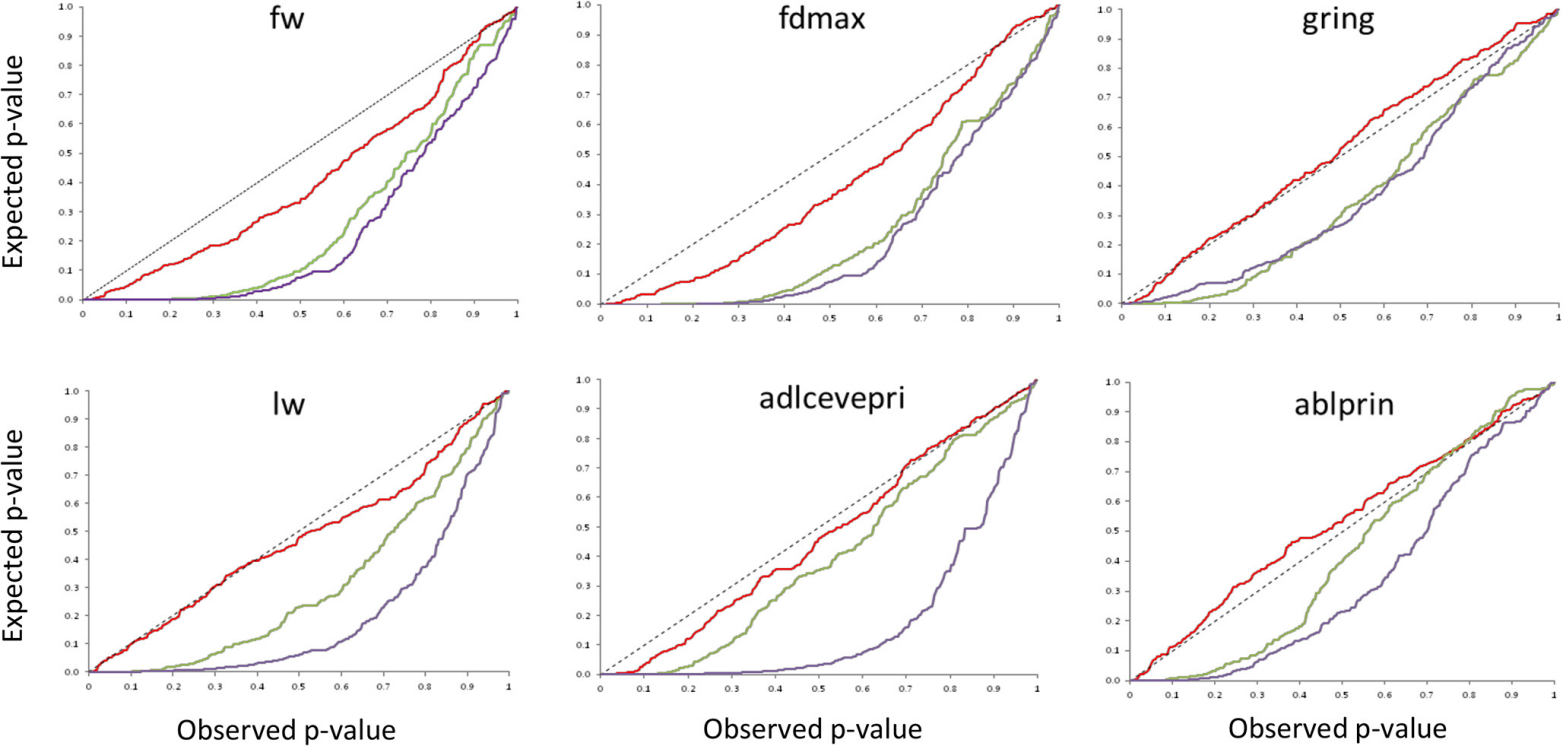
Cumulative density functions based on the three alternative GWA models. The GLM Naive (violet trace), GLM Q-model (green trace) and MLM (red trace). Traits showing significant associations and providing the most consistent p-values are indicated.

**Fig 2:**
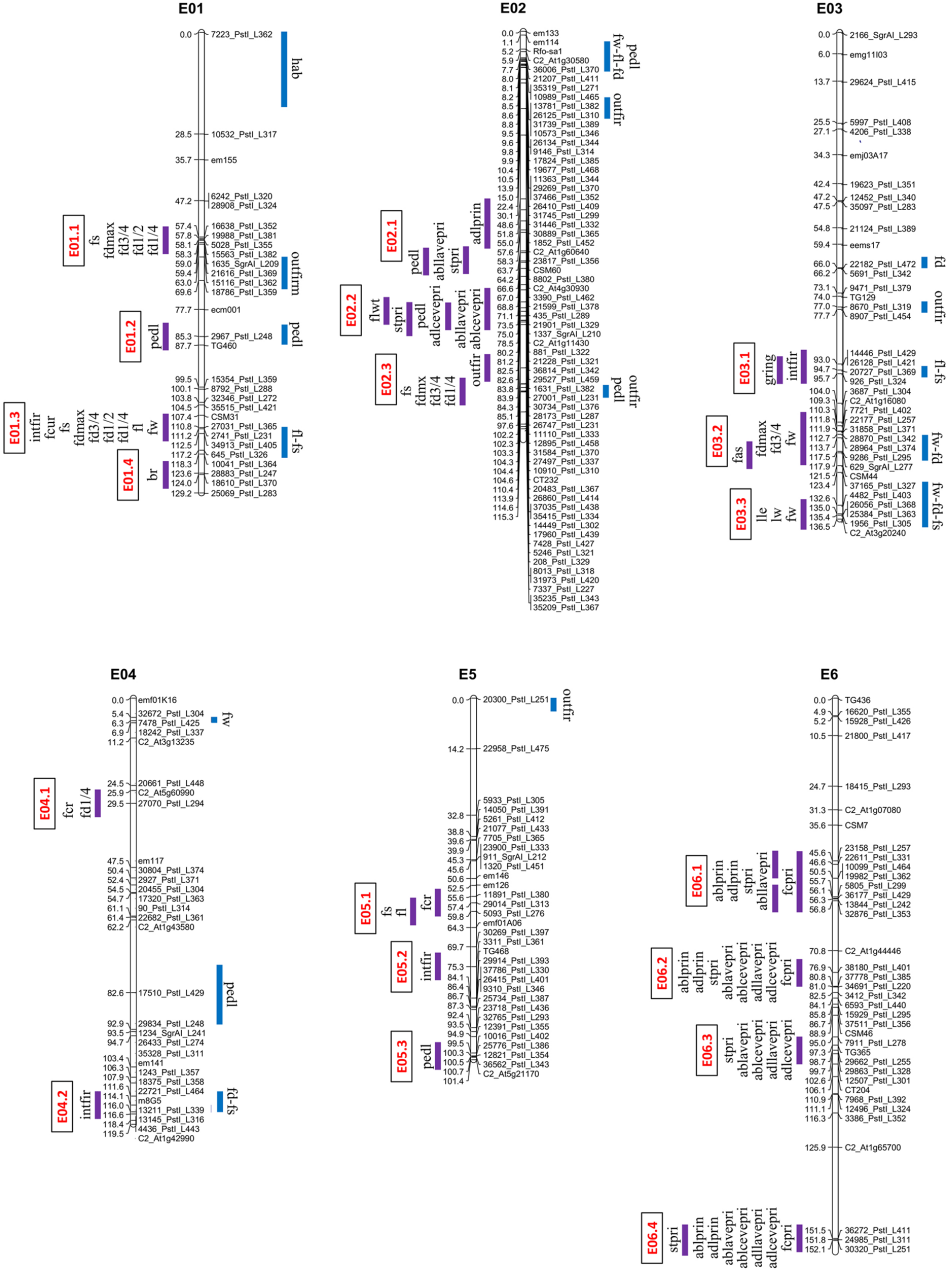
Regions identified by GWA in eggplant LG E01-E06 in comparison to QTL locations described by Portis et al. [2]. The GWA outcome is given to the left of each chromosome (the vertical bars represent a ±3.4 cM interval around the position of the associated SNP loci) and the various regions are marked in red. The QTL locations are shown to the right of each chromosome.

**Fig 3:**
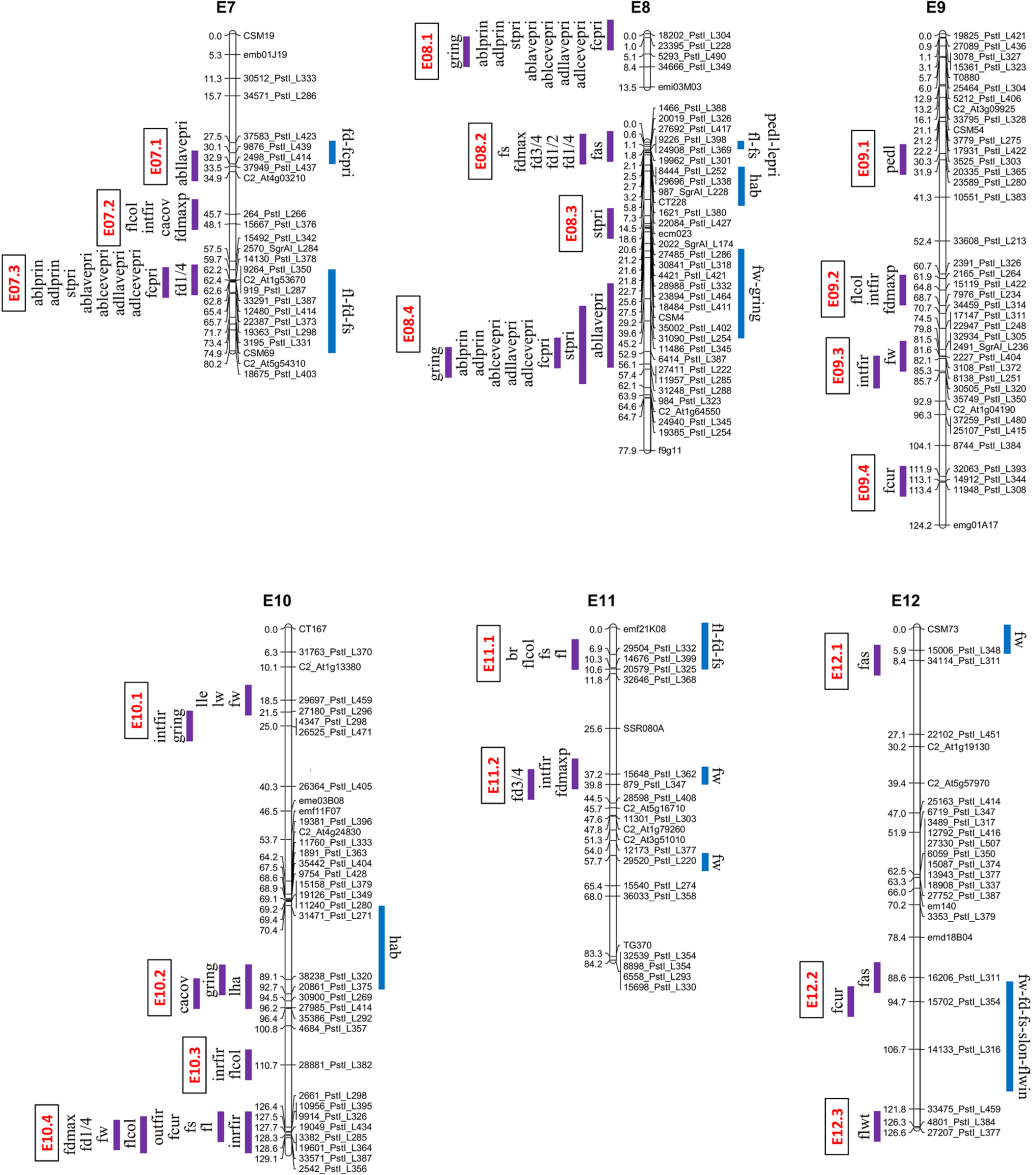
Regions identified by GWA in eggplant LG E07-E12 in comparison to QTL locations described by Portis et al. [2]. The GWA outcome is given to the left of each chromosome (the vertical bars represent a ±3.4 cM interval around the position of the associated SNP loci) and the various regions are marked in red. The QTL locations are shown to the right of each chromosome.

Associations between SNP alleles and fruit/plant/leaf morphology were acquired on the basis of three different models. The GLM Naive-model identified several spurious associations, as suggested by a comparison with probabilities predicted from a theoretical uniform distribution of p-values (Fig 1). A better picture was obtained by applying the GLM Q-model, but only the MLM K+Q-model produced a distribution of p-values comparable to the theoretical one (Fig 1). Subsequent analyses were therefore based on the MLM model. Following q-value correction, 194 significant phenotype/genotype associations were detected. Regions carrying presumed genes/QTL were identified on each of the 12 chromosomes (Table 2, Figs 2 and 3), and involved 30 of the 33 traits (the exceptions were *slon*, *hab* and *flwin*). The number of associations per trait ranged from two (*fcr*, *cacov*, *outfir*, *br*, *lha* and *flwt*) to 17 (*intfir* and *stpri*), with a mean minimum allele frequency (MAF) of 30.7%. Twenty-three markers showed a strong association with at least one trait (p-val < 0.001) and the number went up to 79 when a less stringent threshold was applied (0.05).

**Table 2:** Phenotype/genotype associations uncovered by GWA analysis.

Trait	Marker	Chrom.	Position (cM)*	Association group	p-value	q-value	PVE	MAF
*fw*	27031_PstI_L365	E01	110.78	E01.3	4.73E-05	1.17E-02	6.1%	31.4%
	7721_PstI_L402	E03	110.26	E03.2	8.50E-05	8.30E-03	5.0%	31.4%
	26056_PstI_L368	E03	134.99	E03.3	4.51E-05	8.48E-03	5.5%	45.0%
	1956_PstI_L305	E03	135.44	E03.3	5.63E-05	6.89E-03	5.0%	27.7%
	2227_PstI_L404	E09	81.51	E09.3	2.67E-05	1.75E-03	5.3%	33.5%
	29697_PstI_L459	E10	18.51	E10.1	3.10E-04	1.70E-02	8.2%	6.8%
	19601_PstI_L364	E10	128.34	E10.4	7.49E-04	7.55E-02	5.0%	29.8%
	3382_PstI_L285	E10	128.34	E10.4	7.49E-04	4.11E-02	5.0%	29.8%
	33571_PstI_L387	E10	128.55	E10.4	7.49E-04	4.11E-02	5.0%	29.8%
	9476_PstI_L332	Unmapped	-	-	8.54E-05	1.17E-02	9.0%	14.7%
*fl*	27031_PstI_L365	E01	110.78	E01.3	3.82E-04	2.08E-02	5.0%	31.4%
	5093_PstI_L276	E05	59.81	E05.1	1.89E-04	2.08E-02	7.5%	34.0%
	2661_PstI_L298	E10	126.41	E10.4	4.51E-04	2.47E-02	5.3%	15.2%
	29504_PstI_L332	E11	6.90	E11.1	2.88E-04	2.08E-02	5.3%	36.6%
*fd1/4*	15563_PstI_L382	E01	58.34	E01.1	2.63E-04	2.03E-02	5.0%	23.6%
	27031_PstI_L365	E01	110.78	E01.3	1.02E-05	2.97E-03	6.9%	31.4%
	5578_PstI_L312	Unmapped	-	E02.3*	7.08E-04	3.45E-02	7.2%	16.2%
	27070_PstI_L294	E04	29.54	E04.1	7.95E-04	4.37E-02	5.0%	49.2%
	14130_PstI_L378	E07	62.16	E07.3	5.65E-04	3.30E-02	6.4%	19.4%
	9226_PstI_L398	E08	1.80	E08.2	2.58E-04	2.51E-02	8.1%	19.4%
	19601_PstI_L364	E10	128.34	E10.4	1.89E-05	1.82E-03	6.0%	29.8%
	3382_PstI_L285	E10	128.34	E10.4	1.89E-05	1.04E-03	5.0%	29.8%
	33571_PstI_L387	E10	128.55	E10.4	1.89E-05	1.04E-03	5.0%	29.8%
	9476_PstI_L332	Unmapped	-	-	3.58E-04	2.61E-02	5.0%	14.7%
*fd1/2*	15563_PstI_L382	E01	58.34	E01.1	4.46E-04	3.57E-02	5.0%	23.6%
	27031_PstI_L365	E01	110.78	E01.3	6.16E-06	2.08E-03	6.4%	31.4%
	9226_PstI_L398	E08	1.80	E08.2	2.62E-04	2.95E-02	7.1%	19.4%
	9476_PstI_L332	Unmapped	-	-	4.25E-04	3.59E-02	4.6%	14.7%
*fd3/4*	15563_PstI_L382	E01	58.34	E01.1	1.02E-05	9.61E-04	7.9%	23.6%
	27031_PstI_L365	E01	110.78	E01.3	1.38E-06	4.68E-04	7.2%	31.4%
	17960_PstI_L439	E02	104.33	E02.3	9.08E-04	4.39E-02	7.3%	21.5%
	5578_PstI_L312	Unmapped	-	E02.3*	7.57E-04	4.26E-02	6.4%	16.2%
	7721_PstI_L402	E03	110.26	E03.2	4.87E-04	3.30E-02	7.4%	31.4%
	9226_PstI_L398	E08	1.80	E08.2	4.88E-04	3.30E-02	6.4%	19.4%
	879_PstI_L347	E11	39,78	E11.2	1.58E-05	8.67E-04	5.3%	47.1%
	9476_PstI_L332	Unmapped	-	-	3.70E-04	3.30E-02	8.1%	14.7%
*fdmax*	15563_PstI_L382	E01	58.34	E01.1	4.55E-04	5.05E-02	5.0%	23.6%
	27031_PstI_L365	E01	110.78	E01.3	2.69E-06	9.09E-04	6.6%	31.4%
	5578_PstI_L312	Unmapped	-	E02.3*	8.76E-04	4.94E-02	7.2%	16.2%
	7721_PstI_L402	E03	110.26	E03.2	5.71E-04	3.86E-02	7.3%	31.4%
	9226_PstI_L398	E08	1.80	E08.2	2.19E-04	2.47E-02	7.4%	19.4%
	19601_PstI_L364	E10	128.34	E10.4	5.09E-04	3.99E-02	5.3%	29.8%
	3382_PstI_L285	E10	128.34	E10.4	6.14E-05	3.37E-03	5.3%	29.8%
	33571_PstI_L387	E10	128.55	E10.4	5.09E-04	2.80E-02	5.3%	29.8%
	9476_PstI_L332	Unmapped	-	-	5.21E-04	3.86E-02	8.0%	14.7%
*fdmaxp*	264_PstI_L266	E07	45.75	E07.2	4.47E-04	4.36E-02	6.0%	33.0%
	15119_PstI_L422	E09	64.75	E09.2	6.23E-04	3.92E-02	5.3%	39.8%
	15648_PstI_L362	E11	37.20	E11.2	1.58E-04	1.93E-02	5.0%	33.0%
*fs*	15563_PstI_L382	E01	58.34	E01.1	2.21E-04	2.69E-02	5.8%	23.6%
	27031_PstI_L365	E01	110.78	E01.3	1.98E-05	6.12E-03	8.5%	31.4%
	17960_PstI_L439	E02	104.33	E02.3	4.15E-04	3.21E-02	5.8%	21.5%
	5093_PstI_L276	E05	59.81	E05.1	3.38E-04	1.85E-02	5.0%	34.0%
	9226_PstI_L398	E08	1.80	E08.2	3.42E-04	3.21E-02	5.9%	36.2%
	2661_PstI_L298	E10	126.41	E10.4	4.81E-04	2.64E-02	5.2%	15.2%
	29504_PstI_L332	E11	6.90	E11.1	9.20E-04	8.98E-02	5.0%	36.6%
*fas*	9286_PstI_L295	E03	117.54	E03.2	4.38E-05	8.24E-03	10.2%	19.4%
	27692_PstI_L417	E08	1.14	E08.2	1.20E-04	4.05E-02	9.0%	39.8%
	34114_PstI_L311	E12	8.37	E12.1	1.86E-04	3.50E-02	9.8%	44.5%
	16206_PstI_L311	E12	88.56	E12.2	3.05E-04	3.66E-02	7.4%	33.7%
*fcur*	27031_PstI_L365	E01	110.78	E01.3	8.11E-05	1.49E-02	7.5%	31.4%
	14912_PstI_L344	E09	113.15	E09.4	1.53E-04	1.49E-02	6.9%	30.4%
	2661_PstI_L298	E10	126.41	E10.4	3.76E-04	2.06E-02	6.0%	15.2%
	15702_PstI_L354	E12	94.71	E12.2	1.48E-04	1.49E-02	8.5%	19.9%
*pedl*	2967_PstI_L248	E01	85.28	E01.2	1.6E-05	8.77E-04	6.7%	47.1%
	21228_PstI_L321	E02	66.95	E02.1	6.73E-04	4.66E-02	7.4%	24.6%
	12895_PstI_L458	E02	82.62	E02.2	9.40E-04	4.80E-02	7.1%	38.7%
	12821_PstI_L354	E05	100.51	E05.3	2.28E-04	1.14E-02	8.7%	44.5%
	36562_PstI_L343	E05	100.67	E05.3	1.39E-04	7.65E-03	6.2%	42.9%
	23589_PstI_L280	E09	31.92	E09.1	2.50E-04	4.47E-02	8.4%	22.0%
*fcpri*	22611_PstI_L331	E06	46.57	E06.1	1.04E-03	3.27E-02	4.6%	27.2%
	10099_PstI_L464	E06	50.48	E06.1	8.14E-04	3.27E-02	7.1%	33.0%
	13844_PstI_L242	E06	56.27	E06.1	1.08E-03	3.27E-02	4.5%	32.5%
	38180_PstI_L401	E06	76.91	E06.2	2.10E-05	3.49E-03	7.7%	48.7%
	36272_PstI_L411	E06	151.48	E06.4	2.56E-05	3.49E-03	7.4%	49.2%
	12480_PstI_L414	E07	62.81	E07.3	1.36E-03	3.64E-02	8.0%	34.6%
	23395_PstI_L228	E08	1.03	E08.1	1.08E-04	9.85E-03	6.7%	19.4%
	6414_PstI_L387	E08	52.87	E08.4	1.47E-03	3.64E-02	8.1%	39.8%
*fcr*	27070_PstI_L294	E04	29.54	E04.1	1.10E-04	3.71E-02	7.7%	49.2%
	29014_PstI_L313	E05	57.38	E05.1	5.40E-04	5.27E-02	5.0%	20.9%
*cacov*	264_PstI_L266	E07	45.75	E07.2	1.01E-04	9.89E-03	5.7%	33.0%
	20861_PstI_L375	E10	92.7	E10.2	1.90E-04	1.23E-02	6.2%	38.2%
*outfir*	20483_PstI_L367	E02	97.62	E02.3	9.11E-04	5.75E-02	5.2%	49.2%
	2661_PstI_L298	E10	126.41	E10.4	7.16E-04	3.93E-02	5.8%	15.2%
*intfir*	27031_PstI_L365	E01	110.78	E01.3	1.46E-03	3.58E-02	5.2%	31.4%
	14446_PstI_L429	E03	92.97	E03.1	1.52E-03	3.58E-02	6.7%	31.9%
	26128_PstI_L421	E03	92.97	E03.1	1.45E-03	3.58E-02	6.7%	33.0%
	20727_PstI_L369	E03	94.67	E03.1	1.59E-03	3.58E-02	6.6%	36.1%
	22721_PstI_L464	E04	114.14	E04.2	1.04E-05	2.18E-03	11.5%	27.7%
	3311_PstI_L361	E05	75.30	E05.2	1.07E-03	3.53E-02	7.0%	35.6%
	264_PstI_L266	E07	45.75	E07.2	1.33E-04	7.48E-03	9.1%	33.0%
	15119_PstI_L422	E09	64.75	E09.2	1.29E-05	2.18E-03	11.3%	39.8%
	30505_PstI_L320	E09	85.28	E09.3	2.82E-04	2.76E-02	6.0%	42.9%
	35749_PstI_L350	E09	85.66	E09.3	2.82E-04	5.30E-02	6.0%	42.9%
	26525_PstI_L471	E10	24.96	E10.1	1.15E-03	3.53E-02	7.0%	35.6%
	28881_PstI_L382	E10	110.73	E10.3	3.31E-04	1.40E-02	8.2%	16.2%
	2661_PstI_L298	E10	126.41	E10.4	1.19E-04	7.48E-03	9.2%	15.2%
	9914_PstI_L326	E10	127.53	E10.4	1.14E-03	3.53E-02	7.0%	18.8%
	19049_PstI_L434	E10	127.73	E10.4	2.60E-05	2.93E-03	10.6%	23.6%
	2542_PstI_L356	E10	129.08	E10.4	9.87E-05	7.48E-03	9.3%	19.9%
	15648_PstI_L362	E11	37.20	E11.2	1.88E-04	9.08E-03	8.7%	33.0%
*flcol*	264_PstI_L266	E07	45.75	E07.2	2.73E-05	2.66E-03	5.0%	33.0%
	15119_PstI_L422	E09	64.75	E09.2	3.26E-06	1.99E-04	5.3%	39.8%
	28881_PstI_L382	E10	110.73	E10.3	9.74E-08	1.65E-05	15.9%	16.2%
	9914_PstI_L326	E10	127.53	E10.4	4.09E-06	4.61E-04	12.5%	18.8%
	19049_PstI_L434	E10	127.73	E10.4	1.85E-08	6.26E-06	17.3%	23.6%
	2542_PstI_L356	E10	129.08	E10.4	6.62E-06	5.59E-04	12.0%	19.9%
	29504_PstI_L332	E11	6.90	E11.1	8.04E-05	5.44E-03	9.6%	36.6%
*gring*	20727_PstI_L369	E03	94.67	E03.1	8.05E-04	4.71E-02	7.3%	36.1%
	5293_PstI_L490	E08	5.09	E08.1	8.90E-05	1.00E-02	14.1%	6.8%
	11957_PstI_L285	E08	56.12	E08.4	7.36E-05	1.00E-02	9.6%	17.3%
	4347_PstI_L298	E10	24.96	E10.1	5.06E-04	4.94E-02	7.2%	23.0%
	38238_PstI_L320	E10	89.07	E10.2	9.70E-05	1.00E-02	9.4%	21.5%
*br*	18610_PstI_L370	E01	124.05	E01.4	8.90E-05	3.01E-02	5.1%	47.6%
	29504_PstI_L332	E11	6.90	E11.1	5.79E-05	5.65E-03	6.7%	36.6%
*lw*	26056_PstI_L368	E03	134.99	E03.3	9.29E-05	1.40E-02	6.8%	45.0%
	1956_PstI_L305	E03	135.44	E03.3	2.99E-05	9.02E-03	6.9%	27.7%
	29697_PstI_L459	E10	18.51	E10.1	3.55E-04	1.95E-02	8.1%	6.8%
*lle*	26056_PstI_L368	E03	134.99	E03.3	4.04E-06	3.69E-04	8.9%	45.0%
	1956_PstI_L305	E03	135.44	E03.3	1.29E-06	2.78E-04	9.1%	27.7%
	29697_PstI_L459	E10	18.51	E10.1	3.00E-05	1.65E-03	10.4%	6.8%
*adlcevepri*	12895_PstI_L458	E02	82.62	E02.2	1.46E-04	9.87E-03	8.7%	38.7%
	38180_PstI_L401	E06	76.91	E06.2	1.22E-10	2.07E-08	21.2%	48.7%
	29662_PstI_L255	E06	98.72	E06.3	9.53E-04	4.60E-02	6.9%	22.0%
	36272_PstI_L411	E06	151.48	E06.4	4.37E-11	1.48E-08	21.9%	49.2%
	12480_PstI_L414	E07	62.81	E07.3	1.28E-04	9.87E-03	8.9%	34.6%
	23395_PstI_L228	E08	1.03	E08.1	1.59E-03	4.89E-02	6.5%	19.4%
	6414_PstI_L387	E08	52.87	E08.4	1.38E-04	9.87E-03	8.8%	39.8%
*adllavepri*	38180_PstI_L401	E06	76.91	E06.2	1.22E-18	1.82E-35	56.5%	48.7%
	29662_PstI_L255	E06	98.72	E06.3	3.30E-10	2.79E-04	12.1%	22.0%
	36272_PstI_L411	E06	151.48	E06.4	2.44E-34	2.44E-34	55.1%	49.2%
	12480_PstI_L414	E07	62.81	E07.3	4.18E-06	2.83E-04	12.0%	34.6%
	23395_PstI_L228	E08	1.03	E08.1	1.07E-04	6.03E-03	9.1%	19.4%
	6414_PstI_L387	E08	52.87	E08.4	3.16E-06	2.79E-04	12.2%	39.8%
*ablcevepri*	30734_PstI_L376	E02	78.55	E02.2	6.54E-05	4.79E-03	9.5%	48.7%
	11110_PstI_L333	E02	82.51	E02.2	8.43E-04	2.56E-02	7.1%	19.4%
	12895_PstI_L458	E02	82.62	E02.2	8.17E-05	4.79E-03	9.3%	38.7%
	38180_PstI_L401	E06	76.91	E06.2	6.13E-10	8.39E-08	20.0%	48.7%
	29662_PstI_L255	E06	98.72	E06.3	1.95E-03	4.28E-02	6.3%	22.0%
	36272_PstI_L411	E06	151.48	E06.4	6.15E-10	8.39E-08	19.9%	49.2%
	12480_PstI_L414	E07	62.81	E07.3	8.77E-05	4.79E-03	9.3%	34.6%
	23395_PstI_L228	E08	1.03	E08.1	1.68E-03	4.17E-02	6.6%	19.4%
	6414_PstI_L387	E08	52.87	E08.4	2.79E-04	1.09E-02	8.2%	39.8%
*abllavepri*	881_PstI_L322	E02	66.55	E02.1	5.86E-08	1.42E-06	16.0%	25.7%
	30734_PstI_L376	E02	78.55	E02.2	4.52E-08	1.17E-06	16.2%	48.7%
	11110_PstI_L333	E02	82.51	E02.2	1.43E-13	9.67E-12	26.5%	19.4%
	12895_PstI_L458	E02	82.62	E02.2	5.39E-15	5.63E-13	29.0%	38.7%
	22611_PstI_L331	E06	46.57	E06.1	1.60E-03	2.16E-02	6.5%	27.2%
	13844_PstI_L242	E06	56.27	E06.1	1.49E-03	2.10E-02	6.6%	32.5%
	38180_PstI_L401	E06	76.91	E06.2	7.51E-34	7.51E-34	55.5%	48.7%
	29662_PstI_L255	E06	98.72	E06.3	3.95E-06	7.42E-05	12.2%	22.0%
	36272_PstI_L411	E06	151.48	E06.4	1.40E-33	1.40E-33	55.0%	49.2%
	37949_PstI_L437	E07	33.53	E07.1	9.19E-05	1.48E-03	9.3%	26.7%
	12480_PstI_L414	E07	62.81	E07.3	2.62E-07	5.90E-06	14.8%	34.6%
	23395_PstI_L228	E08	1.03	E08.1	1.45E-04	2.23E-03	9.0%	19.4%
	31090_PstI_L254	E08	39.58	E08.4	3.28E-05	5.84E-04	10.2%	35.6%
	11486_PstI_L345	E08	45.23	E08.4	6.50E-04	9.55E-03	7.4%	17.8%
	6414_PstI_L387	E08	52.87	E08.4	3.31E-07	6.99E-06	14.5%	39.8%
*stpri*	881_PstI_L322	E02	66.55	E02.1	2.27E-03	3.16E-02	6.2%	25.7%
	11110_PstI_L333	E02	82.51	E02.2	2.90E-03	3.46E-02	5.9%	19.4%
	12895_PstI_L458	E02	82.62	E02.2	3.39E-03	3.86E-02	5.8%	38.7%
	10910_PstI_L310	E02	84.35	E02.2	2.63E-04	4.49E-03	8.3%	28.3%
	22611_PstI_L331	E06	46.57	E06.1	3.73E-05	1.33E-03	10.1%	27.2%
	13844_PstI_L242	E06	56.27	E06.1	2.49E-04	4.49E-03	8.4%	32.5%
	38180_PstI_L401	E06	76.91	E06.2	6.00E-15	8.76E-13	29.3%	48.7%
	29662_PstI_L255	E06	98.72	E06.3	2.35E-04	4.49E-03	8.4%	22.0%
	36272_PstI_L411	E06	151.48	E06.4	7.00E-15	8.76E-13	29.0%	49.2%
	30320_PstI_L251	E06	152.13	E06.4	2.58E-03	3.38E-02	6.1%	46.6%
	12480_PstI_L414	E07	62.81	E07.3	1.15E-05	5.52E-04	11.3%	34.6%
	23395_PstI_L228	E08	1.03	E08.1	5.49E-05	1.72E-03	9.9%	19.4%
	27485_PstI_L286	E08	20.60	E08.3	2.22E-04	4.49E-03	8.4%	37.2%
	11486_PstI_L345	E08	45.23	E08.4	4.08E-03	4.44E-02	5.6%	17.8%
	6414_PstI_L387	E08	52.87	E08.4	1.32E-05	5.52E-04	11.1%	39.8%
	27411_PstI_L222	E08	56.10	E08.4	1.95E-04	4.49E-03	8.5%	24.6%
	31248_PstI_L288	E08	57.39	E08.4	2.69E-04	4.49E-03	8.2%	24.6%
*adlprin*	3390_PstI_L462	E02	51.78	E02.1	5.25E-04	2.53E-02	5.0%	23.0%
	21901_PstI_L329	E02	58.27	E02.1	1.10E-03	4.49E-02	4.9%	19.9%
	22611_PstI_L331	E06	46.57	E06.1	1.08E-04	1.22E-02	5.6%	27.2%
	13844_PstI_L242	E06	56.27	E06.1	2.93E-04	1.78E-02	4.7%	32.5%
	38180_PstI_L401	E06	76.91	E06.2	7.74E-06	1.31E-03	7.7%	48.7%
	36272_PstI_L411	E06	151.48	E06.4	7.29E-07	2.46E-04	10.8%	49.2%
	12480_PstI_L414	E07	62.81	E07.3	2.17E-04	1.78E-02	4.8%	34.6%
	23395_PstI_L228	E08	1.03	E08.1	1.20E-03	4.49E-02	4.9%	19.4%
	6414_PstI_L387	E08	52.87	E08.4	3.16E-04	1.78E-02	4.7%	39.8%
*ablprin*	22611_PstI_L331	E06	46.57	E06.1	1.75E-05	1.06E-03	5.6%	27.2%
	13844_PstI_L242	E06	56.27	E06.1	2.40E-05	1.21E-03	5.4%	32.5%
	38180_PstI_L401	E06	76.91	E06.2	7.31E-07	1.10E-04	8.6%	48.7%
	36272_PstI_L411	E06	151.48	E06.4	4.75E-07	1.10E-04	9.5%	49.2%
	12480_PstI_L414	E07	62.81	E07.3	1.31E-05	9.85E-04	5.7%	34.6%
	23395_PstI_L228	E08	1.03	E08.1	3.39E-04	1.46E-02	5.2%	19.4%
	6414_PstI_L387	E08	52.87	E08.4	1.15E-05	9.85E-04	5.3%	39.8%
*lha*	38238_PstI_L320	E10	89.07	E10.2	1.71E-04	1.67E-02	8.8%	21.5%
	20861_PstI_L375	E10	92.7	E10.2	1.59E-04	1.09E-02	5.3%	38.2%
*flwt*	26747_PstI_L231	E02	81.19	E02.2	6.66E-04	3.66E-02	8.8%	37.2%
	4801_PstI_L384	E12	126.28	E12.3	5.53E-04	5.40E-02	7.6%	19.4%

The associated SNPs’ ID, genomic location, chromosomal region, PVE (phenotypic variability explained) and MAF (minimum allele frequency) are given.

* Markers with unknown map position were positioned on the eggplant chromosomes using synteny with the tomato genome

The PVE values lay between 5% and 57% (mean 9.2%). To correlate the associations with known QTL, SNP loci separated from another by <6.8 cM (double the global estimate for LD, see Cericola et al. 2014) were considered as a unit, and their genomic location was obtained from the Barchi et al. [15] map. Overall, 39 regions were defined, involving 1–7 SNP loci each. The most prominent trait clusters were found on chromosomes E01, E06, E07, E08 and E10 (Figs 2 and 3). Two loci (5578_PstI_L312 and 9476_PstI_L332) were not positioned in the Barchi et al. [15] map, but it was possible to assign one of them to an association group on the basis of synteny analyses.

A full listing of the phenotype/genotype associations is given in Table 2. The most important regions influencing variation in fruit morphology were E01.3 (*fw*, *fl*, *fs*, *fcur*, *intfir* and the fruit diameter traits) and E10.4 (*fw*, *fl*, *fd1/4*, *fdmax*, *fs*, *fcur*, *outfir*, *intfir* and *flcol*). Two regions, one on chromosome E01 (E01.1) and one in the distal part of E02 (E02.3), were associated with variation for fruit diameter and *fs*. E03.2 was associated with variation for fruit diameter, *fw* and *fas*, while the distally located segment E08.2 harboured genes affecting fruit diameter, *fs and fas*. Four regions of chromosome E06 were associated with variation for prickliness (*adllavepri*, *abllavepri*, *adlcevepri*, *ablcevepri*, *stpri*, *ablprin* and *adlprin*), as were E07.3, E08.1 and E08.4. Genes determining *fd1/4* were also located to E07.3 and those influencing *gring* on E08.1 and E08.4. The most informative SNP related to fruit trait variation was 15119_PstI_L422 (E09.2); its MAF was 39.8% and its PVE with respect to *infir* was 11%. The two most informative SNPs related to prickliness were 36272_PstI_L411 (E06.4) and 38180_PstI_L401 (E06.2); the former had a MAF of 49.2% and a PVE for *adlcevepri* and *adlprin* of 55%, while for the latter, the MAF was 48.7% and the PVE was 20–30% for *adlcevepri*, *ablcevepri* and *stri*, and 55–56% for *adllavepri* and *abllavepri*.

### Synteny with other Solanaceae species and the identification of potential candidate genes

**Fig 4:**
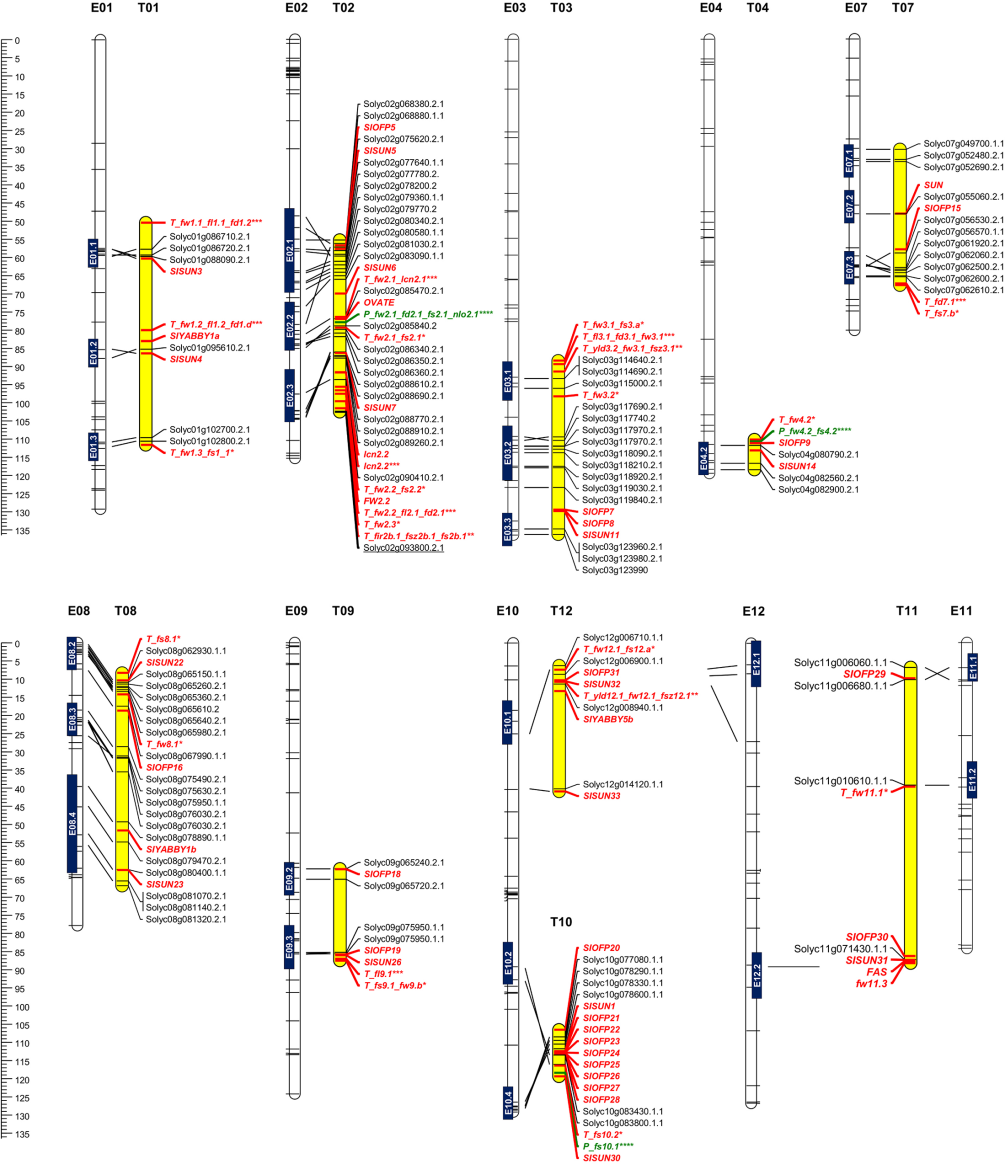
Synteny in the Solanaceae. Eggplant chromosomes are represented by white bars, and the site of QTL detected by GWA analysis is indicated. Tomato chromosomes are represented by yellow bars, along with the position of candidate genes (shown in red). Asterisks indicate orthologous QTL detected in tomato (* Grandillo et al. [30], ** Frary et al. [33], *** Lippman and Tanksley [31]) shown in red, and in pepper (**** Barchi et al. [39], Zygier et al. [38], Chaim et al. [37]) shown in green.

The presence of regions syntenic with either tomato or pepper was identified on ten of the 12 eggplant chromosomes (Fig 4). The regions of chromosome E01 identified with variation for fruit size, weight and shape are orthologous with portions of T01, in which QTL underlying fruit weight, diameter and length, as well as three genes belonging to the *SUN* and *YABBY* gene families have been identified. E02.2 and E02.3 (harbouring factors related to fruit firmness and size) match a portion of T02, in which the fruit shape gene *OVATE*, the fruit weight gene *FW2*.*2* and two *SUN-like* genes have been located, along with QTL underlying fruit weight, size and shape. Synteny have been also evidenced with regions of the pepper genome harboring QTL underlying fruit weight, diameter and shape. On the basis of its homology to the tomato sequence *Solyc02g093800*.*2*.*1* (Fig 3, Table 2), the unmapped SNP marker 5578_PstI_L312 associated with fruit dimensions and weight was placed to E02.3. Regions E03.1 and E03.2, which are associated with variation for *gring*, *intfir*, *fw* and fruit diameter, are syntenic with a portion of T03, in which are located three genes belonging to the *SUN* and *OFP* gene families, along with QTL underlying fruit weight and dimension. The E04.2 region controlling fruit firmness is syntenic with a region of T04 region in which QTL underlying fruit weight and shape, and one member of each the *SUN* and *OFP* gene families have been mapped. The E07.2 region harbouring genes responsible for fruit diameter is syntenic to the segment of the tomato genome in which the fruit shape gene *SUN* has been mapped, while the E07.3 region, the site of genes/QTL underlying both prickliness and fruit diameter, is syntenic to the tomato region in which both an *OVATE*-*like* gene, as well as QTL determining *fs* and fruit diameter have been located. The E08.2 region controlling *fs* and fruit diameter matches the tomato region harbouring two QTL responsible for fruit shape and weight, along with a *SUN-like* gene. The E09.2 and E09.3 regions (fruit weight, firmness and diameter) are syntenic to the portion of T09 carrying QTL for fruit weight, length and shape, as well as three members of the *OFP* and *SUN* families.

The E10.1 region (*fw* and firmness) matches a region of tomato T12, where QTL for fruit weight and shape have been located. The same tomato region harbours a QTL for fruit size and one member of each of the *SUN*, *OFP* and *YABBY* gene families. It also shares synteny with the E12.1 region controlling *fas* and which was previously reported to control eggplant fruit weight [2]. Other regions on E10 (E10.2 and E10.3) match the T10 region harbouring tomato and pepper QTL for fruit shape, two *SUN-like* genes and a cluster of eight *OVATE-like* genes. A tomato *fw* QTL lies in a central portion of T11 syntenic to the E11.2 region, in which QTL controlling fruit diameter and firmness have been mapped. Region E11.1 (fruit length and shape) is syntenic to a different segment of T11 harbouring an *OVATE-like* gene. The T11 gene *FAS*, which is linked to a *fw* QTL and one member of each of the *SUN* and *OFP* gene families, lies in a region syntenic with E12.2, which harbours QTL controlling *fcur* and *fas* and known to control fruit diameter, *fw*, *fs* and *slon* [2].

A search for candidate genes for some of the eggplant QTL was conducted by comparing RAD sequences surrounding the informative SNPs with the tomato genome sequence (S2 Table). The sequence contiguous to the E02.3 SNP 30734_PstI_L376 (linked to *fs*, *fd3/4* and *outfir)* matched a tomato pectinesterase gene (*Solyc02g075620*.*2*.*1*). Within the same E02.3 region, the RAD sequence contiguous to the SNP locus 26747_PstI_L231 matched *Solyc02g085470*.*2*.*1*, a gene encoding a transcription initiation factor, while the 5246_PstI_L321 RAD identified *Solyc02g088690*.*2*.*1* (encoding UDP-glucose 6-dehydrogenase), and the 35415_PstI_L334 RAD identified *Solyc02g089260*.*2*.*1* (encoding an E3 ubiquitin protein ligase). Within E03.3, the RAD sequence associated with 25384_PstI_L363 (linked to *lle*, *lw* and *fw*) identified *Solyc03g123980*.*2*.*1* (encoding a translation repressor). Similarly the E08.2 SNP 987_SgrAI_L228 suggested *Solyc08g065980*.*2*.*1* (encoding a glycine-rich protein) as a candidate for either *fs* and/or fruit size. Finally the RAD sequence contiguous with the E08.4 SNP 6414_PstI_L387 (associated with variation for prickliness and *gring)* matched *Solyc08g080400*.*1*.*1*, a gene encoding a GRAS family transcription factor.

## Discussion

The development of large-scale genotyping capacity has allowed GWA to become a viable approach for the genetic dissection of quantitative traits. Here, the approach has been applied to uncover the genomic regions harbouring genes underlying traits related to fruit, plant and leaf morphology in eggplant.

### Fruit-related trait associations

Eggplant breeding has focused mainly on the improvement of fruit size, weight and shape [23]. Individual fruit size can vary from a few grams to a kilogram but yield tends to correlate with the number of fruits produced by each plant. Moreover, the fruit shape is an important morphological trait selected according with local aesthetic and culinary preferences [24]. In general, fruits with a large calyx and a long peduncle are more desirable. On the other hand calyx prickliness and green pulp are both unattractive features–the former makes handling more difficult and the latter because it gives the impression that the fruit is unripe. Fruit firmness is important both for organoleptic reasons and for the fruits' shelf life. Here, 18 out of the 19 fruit morphology related traits analyzed were associated with markers, a total of 112 trait-markers associations was identified, mapping to 34 genomic regions distributed over all 12 of the eggplant chromosomes. Some of the traits were highly inter-correlated, which implies that many of what appear to be QTL clusters in practice probably represent a single pleiotropic locus.

Some of the traits (notably *outfir*, *intfir* and *cacov*) clearly suffer from a too low level of heritability to allow for effective direct selection by the breeder, while others (notably *fw* and *fs*) showed high levels of heritability. The aggregate PVE associated with each trait was well below the esteemed genetic variance. This problem of “missing heritability” [25] has been apparent ever since molecular markers have been used for genetic analysis. In GWA analysis, the power to detect an association requires a sufficient allele frequency to be present in the germplasm panel, as the signal of a sub-threshold frequency allele (which may have a large effect on the phenotype) may well not be detectable [26]. Here, because the SNPs used were selected from a biparental contrast, a number of polymorphic loci across the association panel were inevitably not assayed. The proposed means of overcoming this weakness is to take a genotyping-by-sequencing (rather than a SNP genotyping) approach, since this would capture all the alleles variants present in the analyzed panel. A second factor accounting for missing heritability is the genetic architecture of the trait. In a large panel of genotypes, variation may be under the control of numerous loci, each of small effect; such loci are hard to be identified above the noise level, unlike those of large effect. A possible approach to address this problem is to increase the density of markers.

A comparison between the genomic locations of a number of phenotype/genotype associations and known QTL is given in Table 3. Some 38% of the markers associated with fruit traits mapped to a region where a relevant QTL has been placed, 18% mapped to a region where an unrelated QTL has been previously located, while 44% were defined genomic regions currently free of any fruit morphology QTL or gene. These latter 15 genomic regions were dispersed over nine chromosomes. Of particular interest are E10.4, which influenced nine fruit traits (*fw*, *fl*, *fd1/4*, *fdmax*, *fs*, *fcur*, *outfir*, *intfir* and *flcol*), E01.1, which harbours factors underlying fruit diameter and *fs*, and E07.2 (fruit diameter, *intfir*, *cacov* and *flcol*) (Fig 3). Further, trait clusters were detected on chromosomes E04, E05 and E09; although some of these await validation, several can be expected to be of interest; these would not have been detected without recourse to an analysis of a wide set of germplasm. Some known QTL were not identified by the GWA analysis, perhaps because the relevant alleles were under- represented in the association panel. In particular, the cluster of QTL determining fruit size and weight mapping to the distal end of chromosome E02 [2] was not identified, presumably because the informative alleles were derived from *S*. *aethiopicum* (introgressed in one parent of the analyzed bi-parental mapping population) rather than from *S*. *melongena*.

**Table 3:** A summary of newly discovered and already established phenotype/genotype associations and QTL/genes controlling morphological variation in eggplant.

Chrom.	Present work		Previous researches	
	Association Group	Trait	QTL/ gene	References
E01	E01.1	*fd1/4; fd1/2; fd3/4; fdmax; fs*	-	-
	E01.2	*pedl*	pedlE01.ML	[2]
	E01.3	*fw*	fw	[6]
		*fl*	flE01.ML	[2]
		*fd1/4; fd1/2; fd3/4; fdmax*	fd1.1	[3]
		*fs*	fsE01.ML; fsE01.MT	[2]
			fs	[6]
		*fcur; intfir*	-	-
	E01.4	*br*	-	-
E02	E02.1	*adlprin*	lp2.1	[4]
		*stpri*	pp2.1	[4]
		*abllavepri; pedl*	-	-
	E02.2	*pedl; adlcevepri; ablcevepri; abllavepr; stpri; flwt*	-	-
	E02.3	*fd1/4; fd3/4; fdmax*	-	-
		*fs*	fs2.1	[3]
		*outfir*	outfirE02b.ML	[2]
E03	E03.1	*intfir; gring*	-	-
	E03.2	*fw*	tyfwE03.ML; eyfwE03.MT	[2]
		*fd3/4*	fd3/4E03.MT	[2]
		*fdmax*	fdmaxE03.ML	[2]
		*fas*	-	-
	E03.3	*fw*	fwE03.ML; fwE03.MT	[2]
		*lw*	-	-
		*lle*	-	-
E04	E04.1	*fd1/4; fcr*	-	-
	E04.2	*intfir*	-	-
E05	E05.1	*fl*	fl	[6]
		*fs; fcr*	-	-
	E05.2	*intfir*	-	-
	E05.3	*pedl*	-	-
E06	E06.1	*fcpri; abllavepri; stpri; ablprin; adlprin*	-	-
	E06.2	*fcpri; adlcevepri; adllavepri;ablcevepri; abllavepri;stpri; ablprin; adlpri*	-	-
	E06.3	*stpri*	sp6.1	[3]
		*adlcevepri; adllavepri;ablcevepri; abllavepri*	-	-
	E06.4	*fcpri*	ftcp6.1; ftcp6.2	[3]; [4]
		*ablprin; adlpri*	lp6.1	[3]
			lp6.2	[4]
		*stpri*	sp6.1; sp6.2	[3]; [4]
			*PRICKLINESS*	[18]
		*adllavepri; abllavepri; adlcevepri; ablcevepri*	-	-
E07	E07.1	*abllavepri*	-	-
	E07.2	*fdmaxp; cacov; intfir; flcol*	-	-
	E07.3	*fd1/4*	fd1/2E07.ML	[2]
			fd	[6]
		*fcpri*	ftcp6.1	[6]
		*adlcevepri; adllavepri; ablcevepri; abllavepri; stpri; ablprin; adlpri*	-	-
E08	E08.1	*fcpri; gring; adlcevepri; adllavepri;ablcevepri; abllavepri;stpri; ablprin; adlpri*	-	-
	E08.2	*fd1/4; fd1/2-; fd3/4; fdmax; fas*	-	-
		*fs*	f2E08.ML	[2]
	E08.3	*stpri*	-	-
	E08.4	*gring*	gringE08ML; gringE08MT	[2]
		*fcpri; adlcevepri; adllavepri; ablcevepri; abllavepri;stpri; ablprin; adlpri*	-	-
E09	E09.1	*pedl*	-	-
	E09.2	*fdmaxp; intfir; flcol*	-	-
	E09.3	*fw*	fw9.1	[3]; [4]
		*intfir*	-	-
	E09.4	*fcur*	-	-
E10	E10.1	*fw; lw; lle; gring; intfir*	-	-
	E10.2	*lha; gring; cacov*	-	-
	E10.3	*intfir; flcol*	-	-
	E10.4	*fw; fl; fd1/4; fdmax; fs; fcur; outfir; intfir; flcol*	-	-
E11	E11.1	*fl*	flE11.MT; flE11.ML	[2]
			fl	[6]
		*fs*	fsE11.MT; fsE11.ML	[2]
		*flcol; br*	-	-
	E11.2	*fd3/4; fdmaxp; intfir*	-	-
E12	E12.1	*fas*	-	-
	E12.2	*fas; fcur*	-	-
	E12.3	*flwt*	-	-

### Plant/leaf morphology-related trait associations

Although prickly types are preferred in certain regions, like Nagpur (India), on the basis of their perceived superior organoleptic quality, prickles are generally considered as undesirable because they can puncture the fruits' skin and are inconvenient in the context of harvesting and storage [27]. Leaf size is an important physiological trait as it has a profound effect on productivity [28], while the upright habit is generally preferred as it may simplify harvesting and cultural practices. Although the number of flowers formed per inflorescence determines the number of fruits set per plant, single flowered inflorescences are generally preferred because they tend to develop larger fruits. Finally, flowering time is a key trait in the context of crop management and can affect the production: shortening the vegetative phase leads to an increase in early yield, and lengthening it may sustain high yield for a long period by the formation of a large number of leaves [29].

The GWA analysis identified phenotype/genotype associations for 12 out of the 15 plant/leaf morphology traits measured. On the whole, 82 traits-markers associations were defined, these were located in 17 distinct genomic regions distributed over nine chromosomes (no associations detected on E04, E05 or E09). Two clusters were identified involving leaf size and several for plant/leaf prickliness. These clusters may comprise a set of closely linked loci or, more likely, they represent a single pleiotropic locus. Several major loci (R2 >0.1) related to prickliness tended to exhibit relatively high heritability, indicating that selection against them would be effective. Only three of the regions associated with prickliness (E02.1, E06.3 and E06.4) matched the site of a known relevant QTL mapped by Doganlar et al. [3] and Frary et al. [5], as well as a trait named *PRICKLINESS*, recently mapped to the distal portion of E06 by Gramazio et al. [18]. Most of the remaining markers (45%) were located in nine genomic regions where no loci related to plant/leaf morphology have been so far been reported, while 36% of the markers mapped to regions already identified as harbouring fruit-related trait loci. In particular, the use of an extensive germplasm has permitted the identification of novel associations with prickliness, notably located on chromosome segments E02.2, E06.1, E06.2 and E08.1 (Figs 2 and 3).

### The use of synteny to identify candidate genes

A substantial effort has been devoted to exploring the mode of inheritance of key fruit traits in the Solanaceae species, predominantly in tomato [4,30–36], but also in pepper [37–39]. Comparative mapping has exposed the high degree of synteny retained between the tomato and eggplant genomes [2,3,15,18,40]. Specifically, the gene content of a genomic region in eggplant harbouring a particular set of trait/marker associations has a good chance of being replicated in the orthologous segments of tomato and pepper. For example, the syntenic regions on E01 and T01 both harbour genes/QTL associated with fruit size, weight and shape [30,31,36], and a similar relationship holds between E08.2 (fruit shape and size) and T08. Synteny-based comparisons between eggplant and tomato were not informative for the genetic basis of plant and leaf morphology, as these traits (e.g., prickliness) are of no relevance to either tomato or pepper. In tomato, fruit weight is under the control of several dispersed genes/QTL [41]. Three genes influencing fruit shape have been isolated, namely *SUN*, *OVATE* and *FAS;* these are members of, respectively, the *IQD/SUN*, *OFP* and *YABBY* gene families [32,42–44]. *OFP* and *YABBY* genes are involved in, respectively, transcriptional repression and lateral organ development, while the function of *IQD/SUN* genes remains unclear [36].

The distal end of the long arm of chromosome T02 harbours the QTL *fw2*.*1*, *fs2*.*1*, *fw2*.*2*, *fs2*.*2*, *fw2*.*3*, *lcn2*.*1*, *lcn2*.*2*, *fl2*.*1*, *fd2*.*1*, *fir2b*.*1*, *fsz2b*.*1* and *fs2b*.*1* [30,31,33], along with *OVATE* and two *SUN-like* genes [36]. The isolation of *fw2*.*2* has shown it to be a gene (*ORFX)*, that is expressed early in floral development and controls carpel cell number [45]. The T02 region is considered to be syntenic to both the eggplant genomic segments E02.3 and E02.4 (the site of genes affecting fruit firmness and size) and the pepper region within which the QTL *fw2*.*1*, *fd2*.*1*, *fs2*.*1* and *nlo2*.*1* all map [37–39], suggesting the involvement of this genomic region, across pepper, eggplant and tomato, in the determination of fruit weight and size. *FAS*, which encodes a transcription factor controlling locule number and thereby fruit weight [43], is tightly linked to the fruit weight QTL *fw11*.*3* [46] and the genes *OFP30* and *SUN31* [36]. The syntenic region in eggplant lies on E12, and harbours QTL underlying the traits *fw*, *fl*, *fd* and *fs* [2]. In addition, the present study has located the control of *fcur* and *fas* to this region, suggesting a shared genetic control of fruit dimensions and fruit curve/apex shape. The T12 region in which *fw12*.*1* and *fs12*. [30], *yld12*.*1*, *fw12*.*1* and *fsz12*.*1* [33] and a member of each of the *SUN*, *OFP* and *YABBY* families [36] matches both E10.1 and E12.1, as a consequence of an evolutionary translocation sequence which differentiates the eggplant and tomato genomes [40]. The E12.1 region, harboring QTL for *fas*, is also the site of a *fw* QTL [2], suggesting shared genetic control of fruit dimensions and apex shape. Fruit shape in tomato is under the joint control of *OVATE* (on T02) [32], *SUN* (T07) [44] and *fs8*.*1* (T08) [47]. An *OVATE* orthologues has been recently mapped on E02 by genotyping an inter-specific population [18]. Here, E02.2, E07.2 and E08.2 were all identified as the sites of genes/QTL influencing fruit diameter and shape. E02.2 is syntenic with the T02 region harbouring *OVATE*, E07.2 with the T07 region harbouring *SUN* and E08.2 with the T08 region harbouring *fs8*.*1* and its proposed candidate gene *SlSUN22* [36] (Fig 4). These syntenic relationships suggest plausible candidate loci for the eggplant genes responsible for fruit shape.

The availability of an annotated tomato genome sequence eases the task of identifying candidate genes for eggplant traits. In the region E02.3 influencing *fs*, *fd3/4* and *outfir*, four candidates have emerged: one encoding a pectinesterase, the second a transcription initiation factor IIB, the third UDP-glucose 6-dehydrogenase and the fourth an E3 ubiquitin-protein ligase (S2 Table). Pectinesterases are expressed in the cell wall and have been implicated in cellular adhesion, stem elongation [48], pollen tube development [49], abscission [50] and fruit ripening [51,52]. The transcription factor IIB may be involved in the expression of a gene/genes controlling fruit dimensions. UDP-glucose 6-dehydrogenase is involved in the synthesis of the cell wall components hemicellulose and pectin, while E3 ubiquitin-protein ligase is essential for auxin efflux and polar auxin transport in such auxin-mediated developmental processes as cell elongation, apical dominance, inflorescence architecture and plant growth and development. A gene encoding a Pumilio RNA binding family protein, located to E03.3 may represent a further plausible candidate for fruit shape and size. This class of proteins is involved with repressing translation [53]. Its *Arabidopsis thaliana* homologues *APUM1*, *APUM2*, *APUM5* and *APUM6* are all highly transcribed in the shoot meristem and newly emerging leaves [54]. Francischini and Quaggio [55] demonstrated that *APUM-1* to *APUM-6* are able to bind specifically to APUM-binding elements in the 3′ UTR of *WUSCHEL*, *CLAVATA-1*, *PINHEAD/ZWILLE* and *FASCIATA-2* transcripts, reported to code for proteins involved in diverse developmental processes, including shoot meristem organization, stem cell maintenance and maintenance of cellular organization of apical meristems [56–60]; WUSCHEL was also identify near the QTL controlling *lc* in tomato [61], and thus we might hypothesized an involvement of Pumilio gene in fruit development. Finally, genes encoding a GRAS family transcription factor and a glycine rich protein emerged as candidates for the control of fruit shape and size. The latter are known to be cell wall constituents, as well as being involved in plant development and cell elongation while the former belongs to a family which includes members involved in silique (i.e. fruit) development in Arabidopsis [62].

## Conclusions

The main objective of the GWA analysis was to elucidate the genetic basis of some key breeding traits in eggplant. The genetic variability captured by the association germplasm panel, which includes contrasting morphology for most of the traits studied here, proved to be a great source of allelic variation. GWA approach successfully validated a number of previously detected QTL, thereby providing the potential for applying a marker assisted selection strategy for improving some key breeders' traits. At the same time, it identified the location of a number of as yet unknown genes/QTL. It was clear that GWA has rather limited power to detect associations for some traits, possibly due to a sub-threshold frequency of functional alleles and/or the presence of many loci each making only a minor contribution to the phenotype. The first problem may be addressable by adopting a genotyping-by-sequencing approach to capture the full range of allelic variation present in the association panel for the targeted sequences. The study has also demonstrated that a comparative genetic approach, relying on the much larger knowledge base associated with tomato, provides an useful short cut for identifying candidate genes. The sequences of such genes can readily provide the materials necessary to develop marker assisted selection assays, while also advancing the understanding of synteny in the Solanaceae. The next step will be to validate the presently identified candidate genes and to identify the allelic variants responsible for trait variation. From a breeding standpoint, the identification of alleles responsible for useful phenotypic variation in eggplant will represent a valuable resource for marker assisted breeding in which combinations of the SNPs explaining the highest fraction of the phenotypic variance can be converted in more easily scorable markers (e.g. HRM, KASP) and used to select the best plant material.

## Supporting Information

S1 Fig PVE values for fruit related traits. Trait codes given in Table 1. Var(g): genotypic variance, Var(m): genotype x location variance, Var(n): genotype x season variance, Var(r): residual variance.
(PDF)

S2 Fig PVE values for plant and leaf related traits. Trait codes given in Table 1. Var(g) = genotypic variance; Var(m) = genotype by location variance; Var(n) = genotype by season variance; Var(r) = residual variance.
(PDF)

S3 Fig Traits related to fruit morphology: Pearson’s inter-trait correlations (upper part of the matrix) and regression coefficients (lower part). The histograms shown on the diagonal illustrate the distribution of trait values (see also Table 1).
(PDF)

S4 Fig Traits related to plant and leaf morphology: Pearson’s inter-trait correlations (upper part of the matrix) and regression coefficients (lower part). The histograms shown on the diagonal illustrate the distribution of trait values (see also Table 1).
(PDF)

S1 Table The 191 eggplant accessions forming the association panel. The provenance of accessions marked “EA” was from east Asia, and that of those marked “WE” from the Mediterranean Basin.
(PDF)

S2 Table BLAST output of eggplant RAD sequences against the tomato genome sequence. (PDF)
