# Supplementary figures and images for: Association Mapping for Fruit, Plant and Leaf Morphology Traits in Eggplant
Source: PLoS One. 2015 Aug 18;10(8):e0135200. doi: 10.1371/journal.pone.0135200 (PMC4540451; doi:10.1371/journal.pone.0135200)

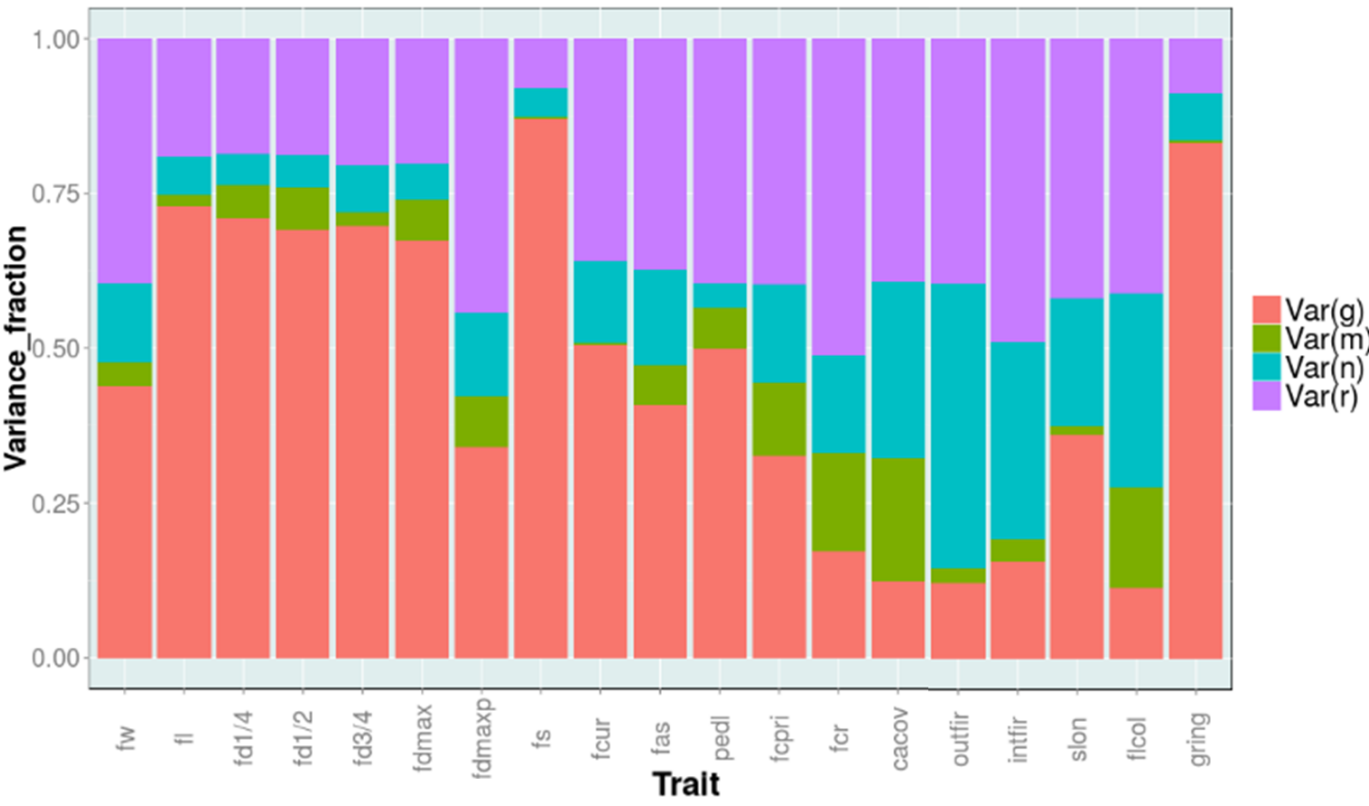

Supplement: S1 Fig — Trait codes given in Table 1. Var(g): genotypic variance, Var(m): genotype x location variance, Var(n): genotype x season variance, Var(r): residual variance. (PDF) [file pone.0135200.s001.pdf]

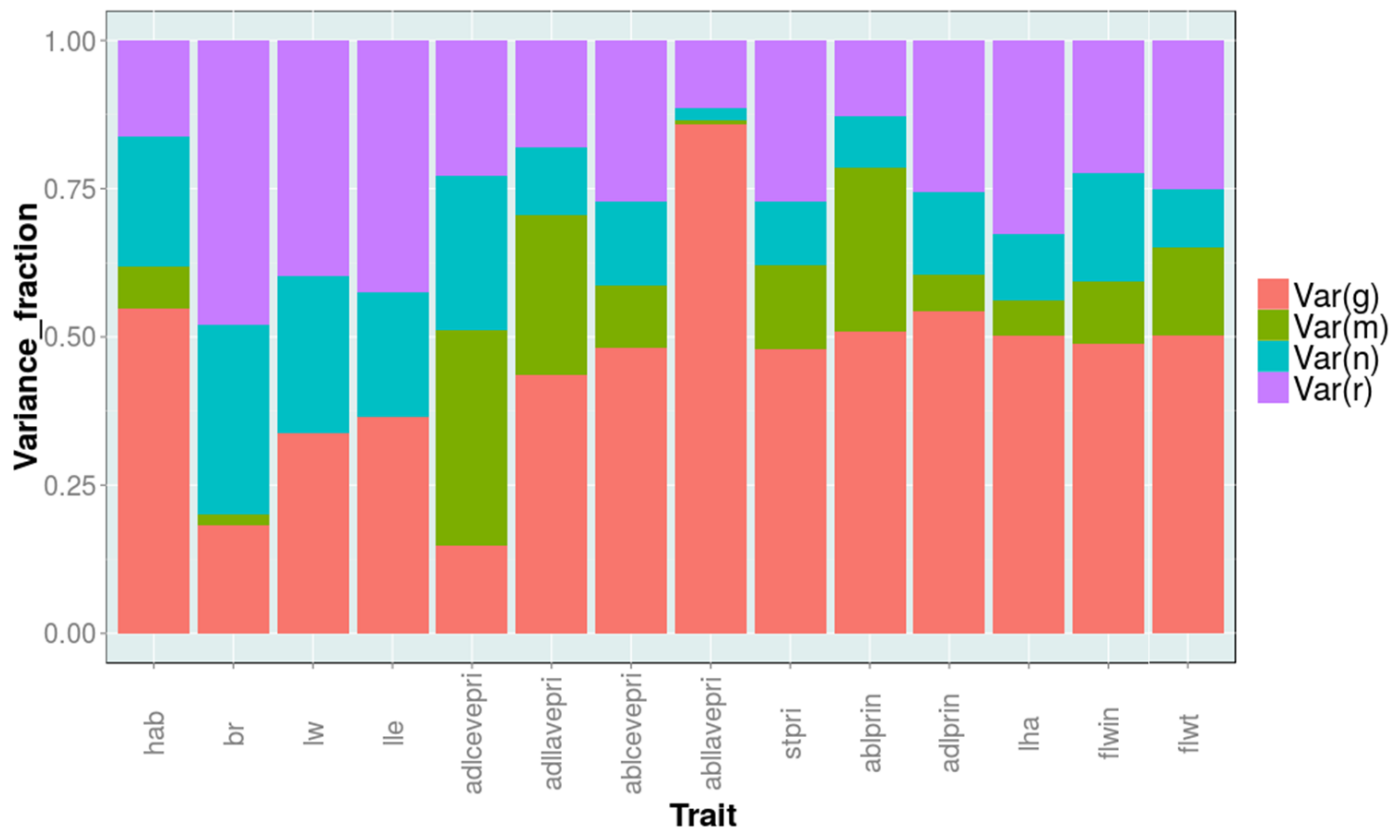

Supplement: S2 Fig — Trait codes given in Table 1. Var(g) = genotypic variance; Var(m) = genotype by location variance; Var(n) = genotype by season variance; Var(r) = residual variance. (PDF) [file pone.0135200.s002.pdf]

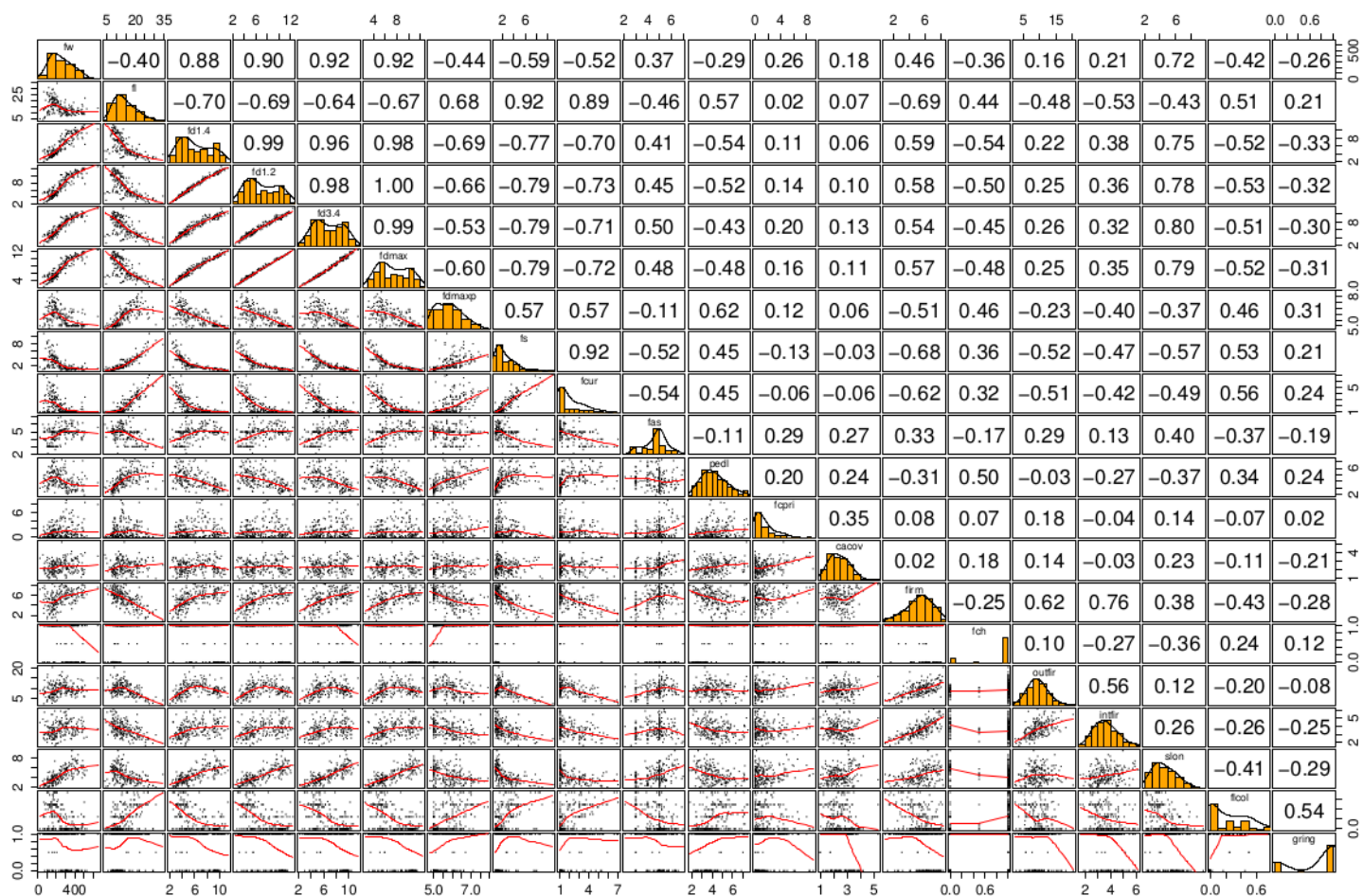

Supplement: S3 Fig — The histograms shown on the diagonal illustrate the distribution of trait values (see also Table 1). (PDF) [file pone.0135200.s003.pdf]

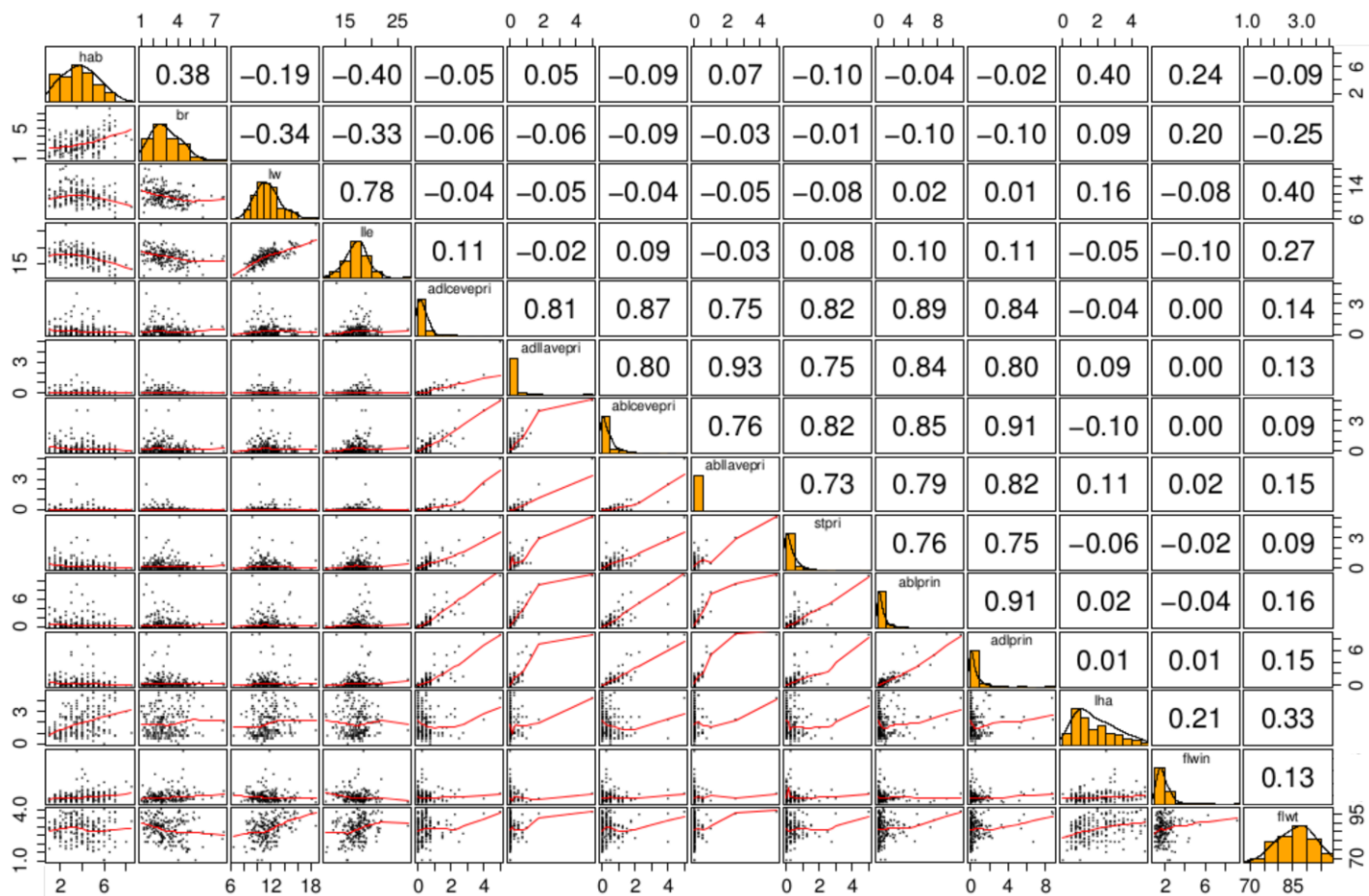

Supplement: S4 Fig — The histograms shown on the diagonal illustrate the distribution of trait values (see also Table 1). (PDF) [file pone.0135200.s004.pdf]
