# Supplementary material for: Association Mapping for Fruit, Plant and Leaf Morphology Traits in Eggplant
Source: PLoS One. 2015 Aug 18;10(8):e0135200. doi: 10.1371/journal.pone.0135200 (PMC4540451; doi:10.1371/journal.pone.0135200)
Supplement: S1 Table — The provenance of accessions marked “EA” was from east Asia, and that of those marked “WE” from the Mediterranean Basin. (PDF) [file pone.0135200.s005.pdf]

Supplementary Table 1 - The 191 eggplant entries forming the association panel. The provenance of entries marked “EA” was from east Asia, and that of those marked “WE” from the Mediterranean Basin.

ID	Accession Name	Origin	Areal	Morphological groups[1]	Structure subpopulations[2]
AM_001	Dadali	Indonesia	EA	1	B
AM_004	Cima viola	Italy	WE	1	A
AM_005	Bianca ovale	Italy	WE	2	B
AM_010	1F5 (9)	Breeding line	WE	2	A
AM_011	Bianca Sicilia	Italy	WE	2	B
AM_013	CCR3	Breeding line	WE	1	A
AM_014	Mel	Italy	WE	2	Admixtured
AM_015	Luga 063	Italy	WE	1	A
AM_016	Prosperosa	Italy	WE	3	B
AM_018	Lunga Violetta Cinese	China	EA	1	B
AM_021	Tal 1/1	Italy	WE	1	A
AM_022	Angiò 4	China	EA	1	B
AM_023	BLK 1269	Breeding line	WE	2	A
AM_024	GIC/ 27-9	Breeding line	WE	2	A
AM_025	Tina	Italy	WE	1	A
AM_026	DR2	Italy	WE	1	A
AM_028	TBE84 D	Breeding line	WE	2	A
AM_029	FanE13 D	Breeding line	WE	2	A
AM_030	FanE27 D	Breeding line	WE	2	A
AM_031	FanE63 D	Breeding line	WE	2	A
AM_032	SNL 534-11	India	EA	3	B
AM_033	SNL 533-8	India	EA	3	B
AM_034	SNL 600-1	India	EA	2	B
AM_035	Cin 01/ 24-6	China	EA	2	B
AM_036	Viola Cin-A-1	China	EA	2	B
AM_037	Violetta di toscana	Italy	WE	3	B
AM_038	Bellezza nera	Italy	WE	2	A
AM_040	Violetta di Metaponto	Italy	WE	3	B
AM_041	28-08/3 (23-09)	Breeding line	WE	3	B
AM_042	31-08/4 (25-09)	Breeding line	WE	3	B
AM_043	51-08/4 (29-09)	Breeding line	WE	3	B
AM_044	52-08/4 (30-09)	Breeding line	WE	3	B
AM_045	55-08/5 (31-09)	Breeding line	WE	3	B
AM_046	16-09 (*15-12)*	Breeding line	WE	3	B
AM_047	P621-08	Breeding line	WE	3	B
AM_048	P623-08	Breeding line	WE	3	B
AM_049	P645-08	Breeding line	WE	3	B
AM_050	P649-08	Breeding line	WE	3	B
AM_051	P612-08	Breeding line	WExEA	3	B
AM_052	P390	Breeding line	WExEA	3	B
AM_053	P328	Breeding line	WExEA	3	B
AM_054	P656-08	Breeding line	WE	3	B
AM_055	msp 73-08	Breeding line	WE	2	A
AM_056	S 1052-08	Breeding line	WE	1	A
AM_057	LI324/06	Italy	WE	1	A
AM_058	msp 36-08	Italy	WE	1	A
AM_059	msp 42-08	Italy	WE	1	A
AM_060	msp 30-08	Italy	WE	1	A
AM_062	msp 55-08	Italy	WE	1	A
AM_063	L422-08	Italy	WE	1	A
AM_064	L717-289	Italy	WE	1	A

AM_067	Uga	Italy	WE	2	A
AM_068	Tana	Italy	WE	1	A
AM_069	Bin 6	Italy	WE	2	A
AM_070	Floralba	Italy	WE	2	Admixtured
AM_071	Ind Min	India	EA	1	Admixtured
AM_072	SM5/2	Breeding line	WE	2	Admixtured
AM_073	SM5/13	Breeding line	WE	2	A
AM_074	SM5/22	Breeding line	WE	1	A
AM_076	S.Nicandro	Italy	WE	2	A
AM_086	LS 3805 minden	Japan	EA	2	B
AM_098	CIN6	China	EA	3	B
AM_099	CIN5	China	EA	3	B
AM_100	CIN7	China	EA	3	B
AM_102	CIN9	China	EA	3	B
AM_103	LS611	Japan	EA	2	Admixtured
AM_106	Naga-Ungu	Indonesia	EA	1	B
AM_114	N 258-4	India	EA	1	Admixtured
AM_121	Indom melanz	Indonesia	EA	2	Admixtured
AM_124	PI17	Italy	WE	2	A
AM_126	Almagro	Spain	WE	2	A
AM_127	Larga negra	Spain	WE	1	A
AM_128	Listada	Spain	WE	2	A
AM_129	Tolga	Algeria	WE	2	A
AM_133	Black Beauty	Italy	WE	2	A
AM_134	Viserba	Italy	WE	1	A
AM_135	Black Beauty	Italy	WE	2	A
AM_136	Tonda Violetta Firenze	Italy	WE	2	B
AM_137	Violetta Lunga Romagna	Italy	WE	1	A
AM_138	Barbentane	France	WE	1	A
AM_139	Lunga Marina	Italy	WE	1	A
AM_140	Tonda di Valence	France	WE	3	B
AM_141	Lunga Violetta Scura Cannellina	Italy	WE	1	A
AM_142	Tonda Black Beauty	Italy	WE	2	A
AM_143	Bellezza Nera	Italy	WE	2	A
AM_144	Lunga Violetta Napoli	Italy	WE	1	A
AM_146	Black Beauty	Italy	WE	2	A
AM_147	Violetta Mostruosa NY	Italy	WE	2	A
AM_148	Slim Jim	India	EA	1	Admixtured
AM_149	Tonda Violetta Scura Valence	France	WE	3	B
AM_150	Grossissima Violetta Firenze	Italy	WE	2	B
AM_151	Violetta Lunga	Italy	WE	1	A
AM_152	Tonda Bianca	Italy	WE	2	Admixtured
AM_153	Prosperosa	Italy	WE	3	A
AM_155	Daejang	China	EA	1	B
AM_156	Buia	Italy	WE	2	A
AM_157	Baffa	Italy	WE	2	A
AM_158	Ank2	India	EA	2	A
AM_159	CN2	China	EA	3	B
AM_160	Dourga	France	WE	2	A
AM_162	Tunisia Baharia	Italy	WE	3	B
AM_163	Pusa Purple Cluster	India	EA	1	Admixtured
AM_167	Angio 3	China	EA	1	B
AM_168	Angio 5	China	EA	2	B
AM_169	Bianca striata verde	Italy	WE	2	Admixtured
AM_170	SM19/14	Breeding line	WE	2	A
AM_171	Palermitana	Italy	WE	3	B

AM_173	Pusa Purple Long	India	EA	1	Admixtured
AM_174	JM (Slim Jim)	India	EA	1	Admixtured
AM_175	Cannellina Sarnense	Italy	WE	1	A
AM_176	Sita	Italy	WE	2	Admixtured
AM_177	FiL white	Turchia	WE	2	Admixtured
AM_178	Lunga napoli	Italy	WE	1	A
AM_179	1237/06	Italy	WE	1	A
AM_180	Listada Tacconi	Italy	WE	2	A
AM_181	Suraj(143)	India	EA	2	Admixtured
AM_182	Pusa Round	India	EA	2	Admixtured
AM_183	Chaojiuye Yuanquie	China	EA	3	B
AM_184	He Shanwang	China	EA	3	B
AM_185	TAI 440	Indochinese Region	EA	3	B
AM_187	Naveen	India	EA	2	Admixtured
AM_188	TAI 444	Indochinese Region	EA	2	Admixtured
AM_189	TAI 445	Indochinese Region	EA	1	Admixtured
AM_190	TAI 446	Indochinese Region	EA	1	Admixtured
AM_191	TAI 449	China	EA	3	B
AM_193	TAI 453	Indochinese Region	EA	2	A
AM_194	TAI 455	Thailand	EA	2	Admixtured
AM_195	TAI 456	Myanmar	EA	2	Admixtured
AM_196	TAI 457	India	EA	2	Admixtured
AM_198	TH 6413 Raos	Indonesia	EA	1	Admixtured
AM_199	TAI 470	Thailand	EA	2	Admixtured
AM_200	TAI 475	Thailand	EA	2	Admixtured
AM_201	TAI 477	Thailand	EA	2	Admixtured
AM_202	TAI 480	India	EA	2	Admixtured
AM_203	TAI 481	China	EA	1	B
AM_204	TAI 483	India	EA	1	Admixtured
AM_205	TAI 484	India	EA	1	Admixtured
AM_206	7 CN	China	EA	3	B
AM_207	9 CN	China	EA	3	B
AM_208	17 CN	China	EA	1	B
AM_210	67-3	Breeding line	WExEA	3	B
AM_211	305 E40	Breeding line	WE	1	A
AM_212	CGN17464 (PI 176759)	Turkey	WE	2	A
AM_213	CGN23345 (PI 169641)	Turkey	WE	2	A
AM_214	CGN18783 (Croisette)	France	WE	1	B
AM_215	CGN18531 (Patchem)	Turkey	WE	1	B
AM_217	CGN17449 (Topak; PI 175917)	Turkey	WE	2	A
AM_218	CGN17451 (Dolmalik; PI 176758)	Turkey	WE	2	A
AM_221	CGN17579 (PI 169648)	Turkey	WE	-	A
AM_222	CGN23346 (Topatan; PI 169649)	Turkey	WE	2	A
AM_224	CGN17581 (PI 169651)	Turkey	WE	1	A
AM_228	CGN23343 (PI 167328)	Turkey	WE	1	A
AM_230	CGN23344 (Bostan; PI 169639)	Turkey	WE	2	A
AM_231	CGN18591 (PI 171847)	Turkey	WE	2	A
AM_232	CGN18595 (PI 171852)	Turkey	WE	1	A
AM_233	CGN18779 (De Barbentane)	France	WE	1	A
AM_234	CGN23309 (Dolg; PI 358232)	Macedonia	WE	1	A
AM_235	CGN18484 (Morska Pata; PI 358242)	Macedonia	WE	1	Admixtured
AM_236	CGN18782 (Violette Longue Hative)	France	WE	1	A
AM_238	CGN17453 (Yesilkoy 27)	Turkey	WE	1	A
AM_240	CGN18578 (Kemer; PI 169655)	Turkey	WE	-	A
AM_241	CGN23348 (PI 169658)	Turkey	WE	1	A
AM_243	CGN18585 (PI 169663)	Turkey	WE	1	A

AM_249	CGN23351 (PI 174362)	Turkey	WE	2	A
AM_251	CGN24467 (Berenjena Listada)	Spain	WE	2	A
AM_252	CGN18505 (Berenjena Redonda)	Spain	WE	3	B
AM_253	CGN24468 (Caminal)	France	WE	1	A
AM_257	CGN18776 (Longue Hative)	France	WE	1	A
AM_258	CGN17456 (Monda)	France	WE	2	Admixtured
AM_259	CGN23315 (Ronde de Valence)	France	WE	3	B
AM_260	CGN17479 (Semiredonda Jaspeada)	Spain	WE	2	A
AM_262	CGN23772	Nigeria	WE	2	Admixtured
AM_264	Mezza Lunga Violetta	Italy	WE	1	A
AM_265	Lunghissima Precoce Violetta	Italy	WE	1	A
AM_266	Dingaras	China	EA	1	B
AM_268	L 129	Indonesia	EA	1	B
AM_269	Talindo	Indonesia	EA	1	B
AM_271	DS1	Breeding line	WE	2	A
AM_273	DS2	Breeding line	WE	2	A
AM_274	DS4	Breeding line	WE	2	A
AM_275	1 CAAS	China	EA	3	B
AM_278	4 CAAS	China	EA	3	B
AM_279	5 CAAS	China	EA	3	B
AM_284	10 CAAS	China	EA	1	A
AM_285	11 CAAS	China	EA	1	B
AM_288	14 CAAS	China	EA	2	A
AM_289	15 CAAS	China	EA	3	B
AM_290	16 CAAS	China	EA	3	B
AM_291	17 CAAS	China	EA	3	B
AM_292	18 CAAS	China	EA	1	B
AM_293	19 CAAS	China	EA	1	B

[1] Morphological groups (1: long, light, curved fruits; 2: oblong fruits of intermediate weight; 3: round, heavy fruits) as defined by Cericola et al. 2013.
[2] Structure subpopulations based on SNP genotyping performed by Cericola et al. 2014. According to the level of membership provided by *STRUCTURE* software, each accession was assigned to a sub-group (A or B) when its level of membership was higher than 70%; The accessions with ambiguous membership were classified as admixed.
