# Supplementary material for: Association Mapping for Fruit, Plant and Leaf Morphology Traits in Eggplant
Source: PLoS One. 2015 Aug 18;10(8):e0135200. doi: 10.1371/journal.pone.0135200 (PMC4540451; doi:10.1371/journal.pone.0135200)
Supplement: S2 Table — (PDF) [file pone.0135200.s006.pdf]

Supplementary Table 2. BLAST corresponding Solyc (*Solanum lycopersicum*) gene sequences and annotation for marker sequences in eggplant association groups

Chrom.	Group	Solyc gene	Annotation result
**E01-T01**	**E01.1**	Solyc01g086710.2.1	DNA-directed RNA polymerase (AHRD V1 ***- B9SVH7_RICCO); contains Interpro domain(s) IPR015712 DNA-directed RNA polymerase, subunit 2
	**E01.1**	Solyc01g086720.2.1	Phosphoribosylanthranilate transferase (Fragment) (AHRD V1 *--- Q43085_PEA); contains Interpro domain(s) IPR013583 Phosphoribosyltransferase C-terminal, plant
	**E01.1**	Solyc01g088090.2.1	Hydrolase alpha/beta fold family protein (AHRD V1 ***- D7M5W5_ARALY); contains Interpro domain(s) IPR000073 Alpha/beta hydrolase fold-1
	**E01.2**	Solyc01g095610.2.1	Leaf senescence protein-like (AHRD V1 ***- Q654T8_ORYSJ); contains Interpro domain(s) IPR004253 Protein of unknown function DUF231, plant
	**E01.3**	Solyc01g102700.2.1	Receptor like kinase, RLK
	**E01.3**	Solyc01g102800.2.1	Histidyl-tRNA synthetase (AHRD V1 *-*- Q52NV4_HUMAN); contains Interpro domain(s) IPR015807 Histidyl-tRNA synthetase, class IIa, subgroup
**E02-T02**	**E02.1**	Solyc02g068380.2.1	N-carbamoyl-L-amino-acid amidohydrolase (AHRD V1 **-- Q1LHR1_RALME); contains Interpro domain(s) IPR010158 Amidase, hydantoinase/carbamoylase
	**E02.1**	Solyc02g068880.1.1	Receptor-interacting serine/threonine-protein kinase 2 (AHRD V1 **** RIPK2_MOUSE); contains Interpro domain(s) IPR002290 Serine/threonine protein kinase
	**E02.1**	Solyc02g075620.2.1	Pectinesterase (AHRD V1 **** B9SZT7_RICCO); contains Interpro domain(s) IPR000070 Pectinesterase, catalytic
	**E02.1**	Solyc02g077640.1.1	Unkonwn
	**E02.1**	Solyc02g077780.2.1	Necrotic spotted lesions 1 (Fragment) (AHRD V1 *-*- B6ZA38_HELPE); contains Interpro domain(s) IPR001862 Membrane attack complex component/perforin/complement C9
	**E02.1**	Solyc02g078200	50S ribosomal protein L21 (AHRD V1 *-*- B6TJJ8_MAIZE); contains Interpro domain(s) IPR001787 Ribosomal protein L21
	**E02.1**	Solyc02g079360.1.1	Octicosapeptide/Phox/Bem1p domain-containing protein (AHRD V1 *--- D7L8F8_ARALY); contains Interpro domain(s) IPR000270 Octicosapeptide/Phox/Bem1p
	**E02.1**	Solyc02g079770.2	DAG protein (AHRD V1 ***- B6TYI4_MAIZE)
	**E02.1**	Solyc02g080340.2.1	Patatin-like phospholipase family protein (AHRD V1 *--* A8PYJ4_BRUMA); contains Interpro domain(s) IPR002641 Patatin
	**E02.1**	Solyc02g080580.1.1	Nuclear protein (AHRD V1 *-*- B6U7S5_MAIZE); contains Interpro domain(s) IPR007608 Protein of unknown function DUF584
	**E02.1**	Solyc02g081030.2.1	Uncharacterized membrane protein C2G11.09 (AHRD V1 **-- YAB9_SCHPO); contains Interpro domain(s) IPR003864 Protein of unknown function DUF221
	**E02.1**	Solyc02g083090.1.1	CM0216.330.nc protein (AHRD V1 ***- B0BLI1_LOTJA); contains Interpro domain(s) IPR004320 Protein of unknown function DUF241, plant
	**E02.2**	Solyc02g085470.2.1	Transcription initiation factor IIB (AHRD V1 *-*- B5XD83_SALSA); contains Interpro domain(s) IPR000812 Transcription factor TFIIB related
	**E02.2**	Solyc02g085840.2	UV excision repair protein RAD23 (AHRD V1 ***- B6JX15_SCHJY); contains Interpro domain(s) IPR000626 Ubiquitin
	**E02.2**	Solyc02g086340.2.1	LisH domain and HEAT repeat-containing protein KIAA1468 (AHRD V1 *-*- K1468_HUMAN); contains Interpro domain(s) IPR011989 Armadillo-like helical
	**E02.2**	Solyc02g086350.2.1	Protein PLASTID MOVEMENT IMPAIRED 2 (AHRD V1 ***- PMI2_ARATH); contains Interpro domain(s) IPR008545 Protein of unknown function DUF827, plant
	**E02.2**	Solyc02g086360.2.1	Octicosapeptide/Phox/Bem1p domain-containing protein (AHRD V1 *--- D7L8F8_ARALY); contains Interpro domain(s) IPR000270 Octicosapeptide/Phox/Bem1p

	**E02.3**	Solyc02g088610.2.1	ATP-dependent chaperone ClpB (AHRD V1 **** B5W0X8_SPIMA); contains Interpro domain(s) IPR017730 Chaperonin ClpB
	**E02.3**	Solyc02g088690.2.1	UDP-glucose 6-dehydrogenase (AHRD V1 **** B6TBY8_MAIZE); contains Interpro domain(s) IPR001732 UDP-glucose/GDP-mannose dehydrogenase, N-terminal
	**E02.3**	Solyc02g088770.2.1	Mitochondrial carrier protein expressed (AHRD V1 ***- Q6AVS9_ORYSJ); contains Interpro domain(s) IPR001993 Mitochondrial substrate carrier
	**E02.3**	Solyc02g088910.2.1	Ninja-family protein 3 (AHRD V1 ***- NNJA3_WHEAT); contains Interpro domain(s) IPR012463 Protein of unknown function DUF1675
	**E02.3**	Solyc02g089260.2.1	E3 ubiquitin-protein ligase UBR4 (AHRD V1 *--- UBR4_HUMAN); contains Interpro domain(s) IPR013993 Zinc finger, N-recognin, metazoa
	**E02.3**	Solyc02g090410.2.1	LOB domain family protein (AHRD V1 ***- D7LG10_ARALY); contains Interpro domain(s) IPR004883 Lateral organ boundaries, LOB
	**E02.3**	Solyc02g093800.2.1	Serine/threonine-protein phosphatase 2A regulatory subunit delta 1 isoform (AHRD V1 ***- Q0CAR1_ASPTN); contains Interpro domain(s) IPR002554 Protein phosphatase 2A, regulatory B subunit, B56
**E03-T03**	**E03.1**	Solyc03g114640.2.1	Signal peptide peptidase SppA type (AHRD V1 ***- Q01NK5_SOLUE); contains Interpro domain(s) IPR004635 Peptidase S49, SppA
	**E03.1**	Solyc03g114690.2.1	Wd-40 repeat-containing protein (AHRD V1 *--- B8C836_THAPS); contains Interpro domain(s) IPR017986 WD40 repeat, region
	**E03.1**	Solyc03g115000.2.1	AT1G74160 protein (Fragment) (AHRD V1 *-*- B9DHP4_ARATH)
	**E03.2**	Solyc03g117690.2.1	Aspartic proteinase nepenthesin-1 (AHRD V1 **-- C0JA51_9ORYZ); contains Interpro domain(s) IPR001461 Peptidase A1
	**E03.2**	Solyc03g117740.2	Chloroplast At1g16080 protein (Fragment) (AHRD V1 *-*- Q1HRY1_SOLLC)
	**E03.2**	Solyc03g117970.2.1	Novel protein similar to vertebrate ADP-ribosyltransferase (NAD+ poly (ADP-ribose) polymerase) (ADPRT) (AHRD V1 **** Q5RHR0_DANRE); contains Interpro domain(s) IPR008288 NAD+ ADP-ribosyltransferase
	**E03.2**	Solyc03g117970.2.1	Novel protein similar to vertebrate ADP-ribosyltransferase (NAD+ poly (ADP-ribose) polymerase) (ADPRT) (AHRD V1 **** Q5RHR0_DANRE); contains Interpro domain(s) IPR008288 NAD+ ADP-ribosyltransferase
	**E03.2**	Solyc03g118090.2.1	Calcium-binding mitochondrial carrier protein SCaMC-1 (AHRD V1 **** B5X2X8_SALSA); contains Interpro domain(s) IPR002113 Adenine nucleotide translocator 1
	**E03.2**	Solyc03g118210.2.1	Os03g0851600 protein (Fragment) (AHRD V1 ***- Q0DLQ8_ORYSJ); contains Interpro domain(s) IPR017986 WD40 repeat, region
	**E03.2**	Solyc03g118920.2.1	ATP-dependent RNA helicase A-like protein (AHRD V1 ***- Q9FF84_ARATH); contains Interpro domain(s) IPR011545 DNA/RNA helicase, DEAD/DEAH box type, N-terminal
	**E03.2**	Solyc03g119030.2.1	Peroxisomal biogenesis factor 3 (AHRD V1 ***- Q28F64_XENTR); contains Interpro domain(s) IPR006966 Peroxin-3
	**E03.2**	Solyc03g119840.2.1	Universal stress protein family protein (AHRD V1 ***- D7KDY5_ARALY); contains Interpro domain(s) IPR006016 UspA
	**E03.3**	Solyc03g123960.2.1	Unknown Protein (AHRD V1)
	**E03.3**	Solyc03g123980.2.1	Pumilio-like protein (AHRD V1 ***- Q9SVZ7_ARATH); contains Interpro domain(s) IPR011989 Armadillo-like helical
	**E03.3**	Solyc03g123990	Mitochondrial carrier protein (AHRD V1 ***- C5P8G6_COCP7); contains Interpro domain(s) IPR002067 Mitochondrial carrier protein
**E04-T04**	**E04.2**	Solyc04g080790.2.1	BEL1-like homeodomain protein 3 (AHRD V1 *--- BLH3_ARATH); contains Interpro domain(s) IPR006563 POX
	**E04.2**	Solyc04g082560.2.1	Actin cytoskeleton-regulatory complex protein PAN1 (AHRD V1 *--- PAN1_MAGGR); contains Interpro domain(s) IPR011992 EF-Hand type
	**E04.2**	Solyc04g082900.2.1	Mitotic checkpoint serine/threonine-protein kinase BUB1 (AHRD V1 *--- BUB1_MOUSE); contains Interpro domain(s) IPR013212 Mad3/BUB1 homology region 1

**E07-T07**	**E07.1**	Solyc07g049700.1.1	Cc-nbs-lrr, resistance protein
	**E07.1**	Solyc07g052480.2.1	Isocitrate lyase (AHRD V1 **** Q9FQD2_IPOBA); contains Interpro domain(s) IPR006254 Isocitrate lyase
	**E07.1**	Solyc07g052690.2.1	Beta-amylase (AHRD V1 **** Q9AT14_CASCR); contains Interpro domain(s) IPR013781 Glycoside hydrolase, subgroup, catalytic core
	**E07.2**	Solyc07g055060.2.1	Phosphoenolpyruvate carboxylase 1 (AHRD V1 ***- O23946_GOSHI); contains Interpro domain(s) IPR015813 Pyruvate/Phosphoenolpyruvate kinase, catalytic core IPR001449 Phosphoenolpyruvate carboxylase
	**E07.3**	Solyc07g056530.2.1	Organic anion transporter (AHRD V1 **-* B6U937_MAIZE); contains Interpro domain(s) IPR004853 Protein of unknown function DUF250
	**E07.3**	Solyc07g056570.1.1	9-cis-epoxycarotenoid dioxygenase
	**E07.3**	Solyc07g061920.2.1	Glucan synthase like 3 (AHRD V1 ***- D8R8I5_SELML); contains Interpro domain(s) IPR003440 Glycosyl transferase, family 48
	**E07.3**	Solyc07g062060.2.1	Peptide methionine sulfoxide reductase msrB (AHRD V1 ***- B6T2N3_MAIZE); contains Interpro domain(s) IPR002579 Methionine sulphoxide reductase B
	**E07.3**	Solyc07g062500.2.1	Cytochrome P450
	**E07.3**	Solyc07g062600.2.1	PHD finger family protein (AHRD V1 *--- D7MI23_ARALY); contains Interpro domain(s) IPR019787 Zinc finger, PHD-finger
	**E07.3**	Solyc07g062610.2.1	4-methyl-5(B-hydroxyethyl)-thiazole monophosphate biosynthesis enzyme ThiJ (AHRD V1 *--- C8T4T4_KLEPR); contains Interpro domain(s) IPR006287 DJ-1
**E08-T08**	**E08.2**	Solyc08g062930.1.1	Calmodulin-binding protein (AHRD V1 **** A2I5F3_BETVU)
	**E08.2**	Solyc08g065150.1.1	Unknown
	**E08.2**	Solyc08g065260.2.1	Ycf36 protein (AHRD V1 *-*- Q55866_SYNY3); contains Interpro domain(s) IPR009631 Protein of unknown function DUF1230
	**E08.2**	Solyc08g065360.2.1	Glutathione-regulated potassium-efflux system protein (AHRD V1 *-** Q8TJ09_METAC); contains Interpro domain(s) IPR006153 Cation/H+ exchanger
	**E08.2**	Solyc08g065610.2	Vacuolar processing enzyme-1b (AHRD V1 **-- Q852T2_TOBAC); contains Interpro domain(s) IPR001096 Peptidase C13, legumain
	**E08.2**	Solyc08g065640.2.1	Thioredoxin-like protein (AHRD V1 **-- Q2UPJ7_ASPOR); contains Interpro domain(s) IPR010400 Protein of unknown function DUF1000
	**E08.2**	Solyc08g065980.2.1	Glycine-rich protein (AHRD V1 ***- D7M4B5_ARALY)
	**E08.2**	Solyc08g067990.1.1	Subtilisin-like protease (AHRD V1 **-- B6SZ82_MAIZE); contains Interpro domain(s) IPR015500 Peptidase S8, subtilisin-related
	**E08.3**	Solyc08g075490.2.1	Carotenoid cleavage dioxygenase 4B
	**E08.3**	Solyc08g075630.2.1	Cc-nbs-lrr, resistance protein
	**E08.3**	Solyc08g075950.1.1	Growth-regulating factor 3 (AHRD V1 **-- B9VHC2_ORYSI); contains Interpro domain(s) IPR014977 WRC
	**E08.3**	Solyc08g076030.2.1	WD-40 repeat protein (AHRD V1 *--- C7RS72_ACCPU); contains Interpro domain(s) IPR017986 WD40 repeat, region
	**E08.3**	Solyc08g076030.2.1	WD-40 repeat protein (AHRD V1 *--- C7RS72_ACCPU); contains Interpro domain(s) IPR017986 WD40 repeat, region
	**E08.4**	Solyc08g078890.1.1	At2g10940-like protein (Fragment) (AHRD V1 *-*- B3SKI0_ARAHA); contains Interpro domain(s) IPR013770 Plant lipid transfer protein and hydrophobic protein, helical
	**E08.4**	Solyc08g079470.2.1	tRNA-modifying protein ygfZ (AHRD V1 *-*- YGFZ_SALTY); contains Interpro domain(s) IPR017703 Folate-binding, YgfZ
	**E08.4**	Solyc08g080400.1.1	GRAS family transcription factor (Fragment) (AHRD V1 *--* B1Q3B1_BRACM); contains Interpro domain(s) IPR005202 GRAS transcription factor

	**E08.4**	Solyc08g081070.2.1	Mitochondrial carrier family (AHRD V1 *-*- C1MZ50_MICPS); contains Interpro domain(s) IPR001993 Mitochondrial substrate carrier
	**E08.4**	Solyc08g081140.2.1	Myc-like anthocyanin regulatory protein (AHRD V1 ***- Q675M5_9ASTE); contains Interpro domain(s) IPR001092 Basic helix-loop-helix dimerisation region bHLH
	**E08.4**	Solyc08g081320.2.1	AP-1 complex subunit beta-1 (AHRD V1 ***- C5GH51_AJEDR); contains Interpro domain(s) IPR016342 Adaptor protein complex, beta subunit
**E09-T09**	**E09.2**	Solyc09g065240.2.1	Patatin-like protein 3 (AHRD V1 ***- B6TPQ5_MAIZE); contains Interpro domain(s) IPR002641 Patatin
	**E09.2**	Solyc09g065720.2.1	Peptidyl-prolyl cis-trans isomerase (AHRD V1 *-*- B9H8E5_POPTR); contains Interpro domain(s) IPR002130 Peptidyl-prolyl cis-trans isomerase, cyclophilin-type
	**E09.3**	Solyc09g075950.1.1	Heat shock protein 1 (AHRD V1 ***- B6SXY0_MAIZE); contains Interpro domain(s) IPR013126 Heat shock protein 70
	**E09.3**	Solyc09g075950.1.1	Heat shock protein 1 (AHRD V1 ***- B6SXY0_MAIZE); contains Interpro domain(s) IPR013126 Heat shock protein 70
**E10-T10**	**E10.4**	Solyc10g077080.1.1	Peroxidase 1 (AHRD V1 ***- A0SWU6_SESRO); contains Interpro domain(s) IPR002016 Haem peroxidase, plant/fungal/bacterial
	**E10.4**	Solyc10g078290.1.1	Alpha-L-arabinofuranosidase (AHRD V1 ***- Q76LU4_SOLLC); contains Interpro domain(s) IPR017853 Glycoside hydrolase, catalytic core
	**E10.4**	Solyc10g078330.1.1	BZIP family transcription factor (Fragment) (AHRD V1 *-*- Q677A7_HYAOR); contains Interpro domain(s) IPR011616 bZIP transcription factor, bZIP-1
	**E10.2**	Solyc10g078600.1.1	Cyclin B-like protein (AHRD V1 ***- Q5U8T5_9SOLA); contains Interpro domain(s) IPR014400 Cyclin, A/B/D/E
	**E10.2**	Solyc10g083430.1.1	F-box family protein (AHRD V1 ***- B9MYU0_POPTR)
	**E10.2**	Solyc10g083800.1.1	Pyrimidine-specific ribonucleoside hydrolase (AHRD V1 ***- B6IUL3_RHOCS); contains Interpro domain(s) IPR001910 Inosine/uridine-preferring nucleoside hydrolase
**E10-T12**	**E10.1**	Solyc12g005480.1.1	Targeting protein for Xklp2 containing protein expressed (AHRD V1 *--- Q10Q22_ORYSJ); contains Interpro domain(s) IPR009675 Targeting for Xklp2
	**E10.1**	Solyc12g005580.1.1	Syntaxin (AHRD V1 ***- Q6X9V9_HORVD); contains Interpro domain(s) IPR010989 t-SNARE
	**E10.1**	Solyc12g014120.1.1	Unknown Protein (AHRD V1)
**E11-T11**	**E11.1**	Solyc11g006060.1.1	Homology to unknown gene (AHRD V1 ***- Q00S02_OSTTA)
	**E11.1**	Solyc11g006680.1.1	Pentatricopeptide repeat-containing protein (AHRD V1 ***- D7LY59_ARALY); contains Interpro domain(s) IPR000504 RNA recognition motif, RNP-1
	**E11.2**	Solyc11g010610.1.1	Small conductance mechanosensitive (MscS-family) ion channel (AHRD V1 *-*- Q31G33_THICR); contains Interpro domain(s) IPR006685 Mechanosensitive ion channel MscS
**E12-T11**	**E12.2**	Solyc11g071430.1.1	Cc-nbs-lrr, resistance protein
